# Advance Care Planning, End-of-Life Preferences, and Burdensome Care: A Pragmatic Cluster Randomized Clinical Trial

**DOI:** 10.1001/jamainternmed.2024.6215

**Published:** 2024-12-02

**Authors:** Jennifer L. Wolff, Danny Scerpella, Erin R. Giovannetti, David L. Roth, Valecia Hanna, Naaz Hussain, Jessica L. Colburn, Martha Abshire Saylor, Cynthia M. Boyd, Valerie Cotter, Maura McGuire, Christine Rawlinson, Danetta H. Sloan, Thomas M. Richards, Kathryn Walker, Kelly M. Smith, Sydney M. Dy

**Affiliations:** 1 Johns Hopkins Bloomberg School of Public Health, Baltimore, Maryland; 2 MedStar Health, Columbia, Maryland; 3 Johns Hopkins School of Medicine, Baltimore, Maryland; 4 Johns Hopkins Community Physicians, Baltimore, Maryland; 5 Johns Hopkins University School of Nursing, Baltimore, Maryland; 6 University of Toronto, Toronto, Ontario, Canada; 7 Michael Garron Hospital, Toronto East Health Network, Toronto, Ontario, Canada

## Abstract

**Importance:**

Primary care is a key setting for advance care planning (ACP).

**Objective:**

To test the effects of a multicomponent primary care–based ACP intervention (SHARING Choices) on documented end-of-life preferences and potentially burdensome care at end of life.

**Design, Setting, and Participants:**

This pragmatic cluster randomized clinical trial in primary care practices from 2 health systems was conducted between March 2021 and April 2022. Adults 65 years and older with a scheduled in-person or telehealth visit with clinicians from participating practices were eligible for inclusion.

**Interventions:**

The treatment protocol encompassed an introductory letter from the clinic, access to a designated facilitator trained in ACP, a person-family agenda-setting checklist, shared access to the patient portal, a mailed advance directive, and print education. The control protocol encompassed usual care.

**Main Outcomes and Measures:**

Primary outcomes included (1) new electronic health record–documented end-of-life preferences (advance directive or medical orders for life-sustaining treatment) at 12 months among those without documentation at baseline and (2) receipt of potentially burdensome care within 6 months of death among a subsample of Maryland residents with serious illness who died within 18 months of study entry from health information exchange data.

**Results:**

There were 19 practices in the intervention arm and 32 practices in the control arm. The study included 22 949 patients in the intervention group (13 575 women [59.2%]; mean [SD] age, 73.9 [7.2] years; 1674 [7.3%] with diagnosed dementia) and 41 966 in the control group (25 057 women [59.7%]; mean [SD] age, 74.0 [7.1] years; 3223 [7.9%] with diagnosed dementia). A total of 17 907 patients (27.6%) were Black, 1373 (2.1%) were Hispanic, 40 345 (62.2%) were White, and 5290 (8.2%) were another race (including American Indian or Alaska Native, Asian, and Native Hawaiian or Other Pacific Islander, and missing race). Grant-funded facilitators initiated 17 931 outreach attempts by phone (13 963 [77.9%]) and through the patient portal (3968 [22.1%]) to patients from intervention practices, and 1181 engaged in facilitator-led ACP conversations. New end-of-life preferences were documented for 2190 of 18 314 patients in the intervention arm (12.0%) and 2130 of 32 321 in the control arm (6.6%). This treatment effect was significant for the overall cohort (adjusted odds ratio, 2.15; 95% CI, 2.02-2.30) and present but attenuated for Black patients, those 75 years and older, and those diagnosed dementia. Among 1498 Maryland resident decedents, potentially burdensome care was higher in the intervention group (150 of 521 [28.8%] vs 204 of 977 [20.9%]; adjusted odds ratio, 1.40; 95% CI, 1.08-1.81).

**Conclusions and Relevance:**

In this pragmatic cluster randomized clinical trial, the SHARING Choices intervention increased new documentation of end-of-life preferences but also increased potentially burdensome care at end of life. Mixed findings underscore the importance of comprehensive support for those with serious illness and prioritizing patient-relevant outcomes in ACP interventional research.

**Trial Registration:**

ClinicalTrials.gov Identifier: NCT04819191

## Key Points

**Question:**

Can a primary care–based advance care planning (ACP) initiative increase electronic health record documentation of end-of-life preferences and reduce potentially burdensome care at end of life?

**Findings:**

In this pragmatic cluster randomized clinical trial of 64 915 older patients from 51 primary care practices, 4.6% of patients from intervention practices engaged in facilitator-led ACP. The multicomponent initiative increased new electronic health record–documented end-of-life preferences yet also increased potentially burdensome care at end of life among a subgroup of decedents with serious illness.

**Meaning:**

Mixed findings emphasize the importance of ascertaining patient-relevant outcomes in ACP interventional research.

## Teaser

This pragmatic cluster randomized clinical trial evaluates the effects of a multicomponent primary care–based advance care planning intervention on documented end-of-life preferences and potentially burdensome care at end of life.

## Introduction

Advance care planning (ACP) is a communication process that supports adults at any age or stage of health in understanding and sharing their personal values, life goals, and preferences regarding future medical care.^1^ Primary care is an important setting for ACP given the presence of long-standing trusted relationships and patients’ preferences that their primary care clinicians initiate such conversations.^2,3^ Yet implementation of ACP in primary care can be challenging as it encompasses a complex set of diverse behaviors, actors, workflows, settings, and policies in the context of constrained time and resources.^4,5^

Older adults who are Black and those living with dementia are less likely to engage in ACP^6^ and are at heightened risk for potentially burdensome care at end of life.^2,7,8,9^ Because ACP in the context of dementia is nuanced,^10,11^ embedded interventional trials have largely excluded persons with dementia outside nursing homes.^12^ Little attention has been directed at strategies to support ACP for this population in primary care, which is a common setting of initial diagnosis and ongoing medical management.^13^

We conducted the SHARING Choices pragmatic cluster randomized clinical trial (NCT04819191) to test the effectiveness of a multicomponent ACP intervention among primary care patients 65 years and older, with special attention to key subgroups by race and dementia status for whom ACP less often occurs. We based implementation on systematic reviews of trials of ACP, input from health system partners, and preliminary pilot work at study sites.^14,15,16,17,18,19^ Our hypotheses were that the intervention protocol would lead to increases in electronic health record (EHR) documentation of end-of-life preferences and reductions in receipt of potentially burdensome care at end of life for those who died with serious illness.

## Methods

This pragmatic cluster randomized clinical trial tested the effects of a multicomponent primary care communication intervention in which the practice was the unit of randomization. The study received approval by the Johns Hopkins Medicine Single Institutional Review Board and was overseen by a 3-member data safety and monitoring board. Informed consent was waived by the institutional review board. Details regarding the trial protocol (Supplement 1)^20^ and implementation^19,21,22^ have been reported. We followed the Consolidated Standards of Reporting Trials (CONSORT) reporting guideline for pragmatic trials.^23^

### Setting and Randomization

The SHARING Choices trial was conducted in all eligible primary care practices operated by 2 health systems in the Mid-Atlantic region. We partnered with health systems that predominantly operate in the state of Maryland to ensure availability of regional health information exchange data. Eligible practices had 2 or more clinicians whose combined panel exceeded 400 older adults.

Of 76 candidate practices, 21 were excluded due to insufficient numbers of clinicians or panel size (n = 15) or organizational reasons, such as competing system initiatives or data constraints (n = 6). The remaining 55 practices underwent random assignment stratified by health system to intervention or control in a 1:2 ratio because control practices incurred no study cost and having more practices affords greater precision in study outcome estimates.

We used a covariate-constrained randomization method to achieve balance on selected practice-level characteristics (location, clinic size, patient panel age, and race distribution).^24^ Between October and December 2020, practices randomized to the intervention were informed by health system leaders that they would be participating in a new initiative to improve communication. Practices in the control group were not informed of inclusion in a research trial.

### Participants

Candidate patients were identified from EHRs of intervention and control practices. We included patients who were 65 years and older due to the recognized importance of normalizing ACP at older ages and their eligibility for Medicare-reimbursed ACP. All patients with a scheduled in-person or telehealth visit with clinicians from participating practices were included: initial visit date served as the point of study entry. There was no formal enrollment or consent.

### Intervention

The SHARING Choices intervention was embedded in primary care practices between September 2020 and March 2021 in a phased process involving partnership and input from health system and primary care practice leaders and involved repeated meetings and contacts with clinicians and staff to socialize the study and refine workflows and monitoring. ACP facilitators were grant-funded lay facilitators hired for the trial (n = 5) or licensed clinical staff (n = 2) already employed by partner health systems. Facilitators were certified in the Respecting Choices First Steps ACP curriculum.^25^ Facilitators were trained in the SHARING Choices protocol (concepts, logistics, rationale); communication strategies (patient portal registration and use, patient-family agenda setting, local context of ACP); and knowledge of dementia over 2 half days. Details regarding the implementation of SHARING Choices have been previously described.^19,20,21,22^

From March 2021 through April 2022, eligible patients with a scheduled visit were sent print or electronic correspondence from the primary care practice stating the importance of sharing wishes about their care and describing strategies to improve communication. The correspondence included a person-family agenda-setting checklist,^26,27^ a description of how to register family or a friend for the patient portal,^28,29^ and a blank advance directive (durable medical power of attorney and living will). The correspondence invited patients to use the checklist to prepare for an upcoming visit, share their electronic health information with family or a friend, talk with their team about their wishes, and complete or return a previously completed advance directive to the primary care practice or to schedule an ACP conversation.

Facilitators reached out to patients via phone and secure messaging to encourage scheduling an ACP conversation. They prioritized contacting patients with diagnosed dementia, upcoming annual wellness visits, or lack of EHR-documented advance directives, to align with health system initiatives related to benchmarks for care quality metrics. In parallel, clinicians and case managers were encouraged to refer patients for ACP. Each health system chose a standardized approach, specific to their health system and practice workflows for how the grant-funded ACP facilitators conducted outreach.

ACP conversations occurred by phone, telehealth, or at the practice. Facilitators followed the Respecting Choices structured conversation guide and used motivational interviewing. Facilitators recorded the duration, modality, and content of ACP meetings in the EHR. Advance directive documentation was uploaded to the EHR when completed with the facilitator, brought to a visit, or electronically or physically sent to the practice.

Patients of primary care practices randomized to the control group received usual care.

### Data Sources

Each health system provided practice-level and patient-level EHR and administrative data to the study team. Practice characteristics included location, specialty, numbers of clinicians, and number and characteristics of attributed patients. Patient characteristics included age, sex, race, ethnicity, patient portal registration, diagnoses, EHR-documented end-of-life preferences, and area deprivation index score for place of residence at the Census block level.^30^ Diagnosis codes were extracted from the EHR at study entry and updated at 12 months. Race, ethnicity, and area deprivation index for residence location were extracted from the EHR at baseline. Race and ethnicity were generally entered into the EHR by practice staff, not patient reported.^31^ Dates of death for Maryland residents were obtained from the Maryland Department of Vital Statistics. Dates of hospital-based services and procedure codes from the Maryland Hospital Services Cost Review Commission were assembled for the cohort, deidentified, and provided to the study team by the Maryland–Washington, DC, health information exchange, the Chesapeake Regional Information System for Our Patients (CRISP).

### Outcomes

Our first primary outcome was a composite measure of new EHR-documented end-of-life preferences, encompassing advance directive or Maryland Medical Order for Life-Sustaining Treatmentor District of Columbia Medical Orders for Scope of Treatment from each health system.^32^ Documentation was extracted at baseline and 12 months after study entry. We differentiated new EHR documentation (for those without documentation at baseline) from updated documentation (for those with documentation at baseline). A secondary outcome was new advance directives (vs the composite measure), which was prioritized in our study.

Our second primary outcome was potentially burdensome care at end of life, encompassing procedures with potential for causing harm or prolonging suffering in the context of advanced serious illness (eg, gastrostomy tube insertion, intubation/mechanical ventilation). The numerator included specific procedures using dates and validated *International Statistical Classification of Diseases, Tenth Revision, Clinical Modification *(*ICD*-*10*-*CM*) codes for hospital services.^33,34^ The denominator comprised patients who died with a diagnosis of serious illness, using a modified list of validated *ICD*-*10*-*CM* codes of diagnoses conveying advanced severity.^35^ We focused on deaths within 18 months of study entry and eliminated care preceding intervention launch. This analysis was limited to Maryland residents, for whom dates of death and hospital-based procedures were available. Additional details about the construction of outcomes are provided in eTables 1 and 2 in Supplement 2.

### Masking

This was a pragmatic trial in which intervention processes were included as routine care. Primary care practice group assignment was known to each health system and the study team, including lead statistician and analysts (including D.L.R.) who were involved in randomization and preparing data safety and monitoring reports.

### Statistical Analysis

As the study was designed to produce balance on covariates at the practice level, we assessed the distribution of patient-level characteristics to evaluate comparability by treatment group at baseline. Analyses followed the intention-to-treat principle, with the primary independent variable being group assignment when patients were first identified, including the small number of patients (138 [0.2%]) identified through CRISP as receiving care at both systems.

We built multilevel models accounting for practice-level randomization, group assignment, and clustering of outcome distributions within practices. Multilevel models were performed using the Proc Glimmix package in SAS statistical software version 9.4 (SAS Institute) and accounted for the clustered design, with patient as the unit of analysis clustered within randomized practices.

We conducted stratified analyses by dementia diagnosis, age, sex, and race. Analyses of race were limited to patients who were non-Hispanic Black and White, who were well-represented in our cohort, recognizing racial inequities in documentation of end-of-life preferences and care.^8,9,36^ Hispanic ethnicity was not examined due to greater inaccuracies in EHR documentation^31^ and the small numbers of patients identified as Hispanic in our cohort.

Finally, we descriptively examined variation by intervention primary care practice. For each practice, we examined the proportion of patients with new EHR-documented end-of-life preferences, stratified by whether they did or did not engage in ACP. We were unable to report practice-level variation in potentially burdensome care at end of life due to small numbers of decedents.

Power calculations were guided by published effect sizes^37^ and computed using the Optimal Design software package version 1.76 (Optimal Design),^38,39^ assuming a 2-tailed type 1 error of .025 due to having 2 primary outcomes. We expected a cohort of 87 000 eligible patients, 9200 with a diagnosis of dementia, and 7000 who died within 12 months, from national data.^40,41^

We assumed that 15% of patients would have EHR-documented end-of-life preferences at baseline based on information from partner organization at the time of trial design and a plausible range of 10% (ie, from 5% to 25%) from national estimates,^37^ giving us power of 0.80 to detect an increase from 15% to 21% or greater. We assumed 18% of decedents would receive potentially burdensome care at end of life in the absence of the intervention, with a plausible range of 5% (ie, from 13% to 23%) from national data^41^ providing power of 0.80 to detect a reduction in potentially burdensome care from 18% to 15% for decedents.

All *P* values and 95% CIs were calculated from clustered logistic regression analyses. Significance was set at *P* < .05, and all *P* values were 2-tailed.

## Results

### Primary Care Practices

**Figure 1.**
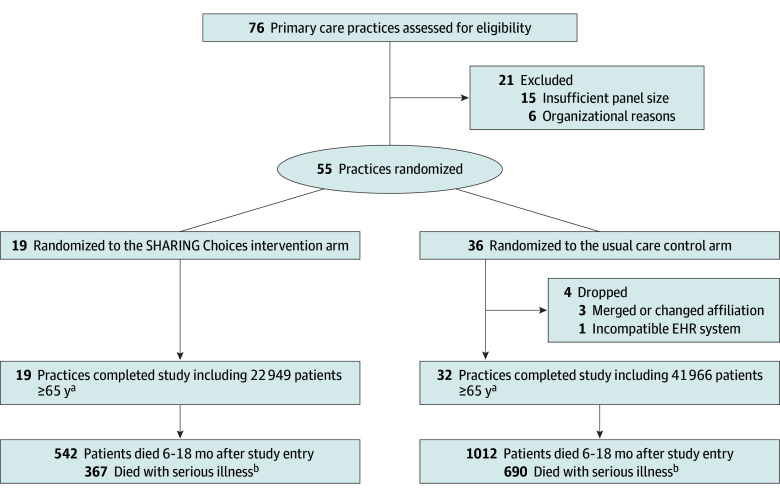
CONSORT Diagram of Primary Care Clinic Randomization A total of 138 patients identified as receiving care at both partner systems were removed from the analytic sample. EHR indicates electronic health record. ^a^Sample for primary outcome analysis of documented advance directive or medical orders for life-sustaining treatment. ^b^Sample for primary outcome analysis of potentially burdensome end-of-life care.

Figure 1 summarizes the study design and participant flow. Of 55 primary care practices that met inclusion criteria for randomization, 19 were allocated to the SHARING Choices intervention and 36 were allocated to the control protocol. After randomization, 4 control practices were dropped for organizational reasons, leaving 32 control practices.

### Participants

The sample for our EHR-documented end-of-life preferences outcome included 22 949 patients from intervention practices (13 575 women [59.2%]; mean [SD] age, 73.9 [7.2] years; 1674 [7.3%] with diagnosed dementia) and 41 966 patients from control practices (25 057 women [59.7%]; mean [SD] age, 74.0 [7.1] years; 3223 [7.9%] with diagnosed dementia) (Table 1). A total of 17 907 patients (27.6%) were Black, 1373 (2.1%) were Hispanic, 40 345 (62.2%) were White, and 5290 (8.2%) were another race (including American Indian or Alaska Native, Asian, and Native Hawaiian or Other Pacific Islander, and missing race). A total of 2271 of 56 951 Maryland residents (4.0%) died during the 18-month observation period, of whom 1498 had a serious illness. The 521 decedents from intervention practices and 977 decedents from control practices composed our cohort for examining potentially burdensome care at end of life.

**Table 1.** Characteristics of Primary Care Practices and Patients at Baseline by Treatment Group

Characteristic	No. (%)		
	SHARING Choices	Control	Total
**Practice characteristics**			
Total, No.	19	32	51
Location			
Urban	5 (26.3)	9 (28.1)	14 (27.4)
Rural	5 (26.3)	5 (15.6)	10 (19.6)
Suburban	9 (47.4)	18 (56.3)	27 (52.9)
Primary care clinicians, No.			
2-4	5 (26.3)	10 (31.3)	15 (29.4)
5-7	10 (52.6)	14 (43.8)	24 (47.1)
6-10	4 (21.0)	8 (25.0)	12 (23.5)
Patients ≥65 y, mean (SD), %	23.5 (9.2)	28.4 (14.5)	26.6 (12.9)
Black patients, mean (SD), %	32.1 (25.2)	29.5 (22.6)	30.5 (23.4)
**Patient characteristics**			
Total, No.	22 949	41 966	64 915
Age, mean (SD), y	73.9 (7.2)	74.0 (7.1)	74.0 (7.1)
Age ≥75 y	9104 (39.7)	17 112 (40.8)	26 216 (40.4)
Sex			
Female	13 575 (59.2)	25 057 (59.7)	38 632 (59.5)
Male	9374 (40.8)	16 909 (40.3)	26 283 (40.5)
Race and ethnicity^a^			
Hispanic	399 (1.7)	974 (2.3)	1373 (2.1)
Non-Hispanic Black	7923 (34.5)	9984 (23.8)	17 907 (27.6)
Non-Hispanic White	12 924 (56.3)	27 421 (65.3)	40 345 (62.2)
Other race	1703 (7.4)	3587 (8.6)	5290 (8.2)
Area deprivation index score, mean (SD)^b^	30.3 (23.2)	24.1 (19.0)	26.3 (20.8)
Dementia diagnosis^c^	1674 (7.3)	3323 (7.9)	4997 (7.7)
Registered for patient portal	11 309 (49.3)	24 966 (59.5)	36 275 (55.9)
State of patient residence			
Maryland	19 571 (85.3)	37 380 (89.1)	56 951 (87.7)
Virginia or Washington, DC	2926 (12.8)	3017 (7.2)	5943 (9.2)
Other	452 (2.0)	1569 (3.7)	2021 (3.1)
**Decedent characteristics** ^d^			
Total, No.	521	977	1498
Age, mean (SD), y	79.8 (8.3)	80.8 (8.4)	80.4 (8.4)
Age ≥75 y	361 (69.3)	734 (75.1)	1095 (73.1)
Sex			
Female	259 (49.7)	537 (55.0)	796 (53.1)
Male	262 (50.3)	440 (45.0)	702 (46.9)
Race and ethnicity^a^^,^^e^			
Non-Hispanic Black	174 (33.4)	258 (26.4)	432 (28.8)
Non-Hispanic White	321 (61.6)	654 (66.9)	975 (65.1)
Hispanic or other race	26 (5.0)	65 (6.7)	91 (6.1)
Area deprivation index score, mean (SD)^b^	36.9 (24.4)	30.7 (22.6)	32.9 (23.5)
Dementia diagnosis^c^	188 (36.1)	418 (42.8)	606 (40.5)
Registered for patient portal	226 (43.4)	447 (45.8)	673 (44.9)

^a^ Race and ethnicity are taken from the electronic health record and were generally entered into the electronic health record by practice staff, not patient reported. The other race category includes American Indian or Alaska Native, Asian, and Native Hawaiian or Other Pacific Islander, and missing race.

^b^ Area deprivation index score ranges from 1 to 100, with higher values reflecting greater deprivation. We used the National Area Deprivation Index.

^c^ Dementia diagnoses are inclusive of diagnoses present at baseline and new diagnoses up to 1 year after study entry.

^d^ Patients with serious illnesses who died 6 to 18 months after study entry.

^e^ Hispanic ethnicity is reported with the other race category due to insufficient cell size to report separately.

### Intervention Processes: Outreach and ACP Conversations

ACP facilitators made 17 931 outreach attempts to intervention practice patients by phone (13 963 [77.9%]) and the patient portal (3968 [22.1%]). A total of 1181 patients from intervention practices (4.6%) engaged in 1 or more ACP conversations during the 12-month observation period. Facilitator-led ACP varied by intervention primary care practice from a low of 49 of 4601 (1.1%) to a high of 234 of 2477 (9.4%) (eFigure 1 in Supplement 2).

### EHR-Documented End-of-Life Preferences

The proportion of patients without EHR-documented end-of-life preferences at baseline who had new documentation at 12 months was 12.0% (2190 of 18 314) in the intervention group and 6.6% (2130 of 32 321) in the control group (adjusted odds ratio [aOR], 2.15; 95% CI, 2.02-2.30) (Table 2). Intervention effects were smaller for updated EHR-documented end-of-life preferences among those with documentation at baseline (aOR, 1.59; 95% CI, 1.43-1.76) and larger for new advance directive documentation (aOR, 3.60; 95% CI, 3.32-3.90) when assessed separately. Among patients with diagnosed dementia, the proportion with newly documented end-of-life preferences was 23.5% (255 of 1087) in the intervention group and 19.6% (409 of 2084) in the control group (aOR, 1.31; 95% CI, 1.09-1.57). The intervention effect was significant for those with updated EHR-documented end-of-life preferences (aOR, 1.57; 95% CI, 1.22-2.02) and somewhat larger for new advance directive documentation (aOR, 1.80; 95% CI, 1.45-2.24).

**Table 2.** Primary Outcomes in the Full Cohort and Intervention Effects by Treatment Group

Outcome	SHARING Choices		Control		OR (95% CI)	aOR (95% CI)^a^
	Total, No.	Events, No. (%)	Total, No.	Events, No. (%)		
**End-of-life preferences documentation in the EHR**						
New advance directive or medical orders for life-sustaining treatment among patients without EHR documentation at baseline^b^^,^^c^						
Full cohort	18 314	2190 (12.0)	32 321	2130 (6.6)	1.93 (1.81-2.05)	2.15 (2.02-2.30)
Older adults with diagnosed dementia	1087	255 (23.5)	2084	409 (19.6)	1.26 (1.05-1.50)	1.31 (1.09-1.57)
New advance directive among patients without EHR documentation at baseline^b^						
Full cohort	20 517	1699 (8.3)	37 471	1010 (2.7)	3.26 (3.01-3.53)	3.60 (3.32-3.90)
Older adults with diagnosed dementia	1364	166 (12.2)	2733	206 (7.5)	1.70 (1.37-2.11)	1.80 (1.45-2.24)
Updated advance directive or medical orders for life-sustaining treatment among patients with EHR documentation at baseline^d^						
Full cohort	4635	710 (15.3)	9645	1060 (11.0)	1.47 (1.32-1.62)	1.59 (1.43-1.76)
Older adults with diagnosed dementia	587	145 (24.7)	1239	197 (15.9)	1.74 (1.36-2.21)	1.57 (1.22-2.02)
**Potentially burdensome care at end of life** ^e^						
Full cohort	521	150 (28.8)	977	204 (20.9)	1.53 (1.20-1.96)	1.40 (1.08-1.81)
Older adults with diagnosed dementia	181	37 (20.4)	409	53 (13.0)	1.73 (1.09-2.74)	1.47 (0.89-2.42)

Abbreviations: aOR, adjusted odds ratio; EHR, electronic health record; OR, odds ratio.

^a^ Adjusted for patient age, sex, race and ethnicity (Hispanic, non-Hispanic Black, non-Hispanic White, and other race), state, and organization.

^b^ New EHR documentation by 12 months among older adults without EHR documentation at baseline.

^c^ Composite documentation refers to either advance directive or medical orders for life-sustaining treatment.

^d^ Updated documentation refers to having an update of documentation of advance directive or medical orders for life-sustaining treatment by 12 months for those with documentation of an advance directive or medical orders for life-sustaining treatment in the EHR at baseline.

^e^ Among Maryland residents with serious illnesses who died 0 to 18 months after study entry.

### Potentially Burdensome Care at End of Life

The percentage of decedents with serious illness who experienced potentially burdensome care within 6 months of death was higher at SHARING Choices practices (150 of 521 decedents [28.8%]) than control practices (204 of 977 decedents [20.9%]). This effect was observed in both unadjusted models (OR, 1.53; 95% CI, 1.20-1.96) and covariate-adjusted models (aOR, 1.40; 95% CI, 1.08-1.81). The percentage of older decedents with diagnosed dementia who experienced potentially burdensome care within 6 months of death was 20.4% (37 of 181 decedents) at SHARING Choices practices and 13.0% (53 of 409 decedents) at control practices. This treatment effect was statistically significant when unadjusted (OR, 1.73; 95% CI, 1.09-2.74) but was not significantly different after covariate adjustment (aOR, 1.47; 95% CI, 0.89-2.42).

### Subgroup Analyses

**Figure 2.**
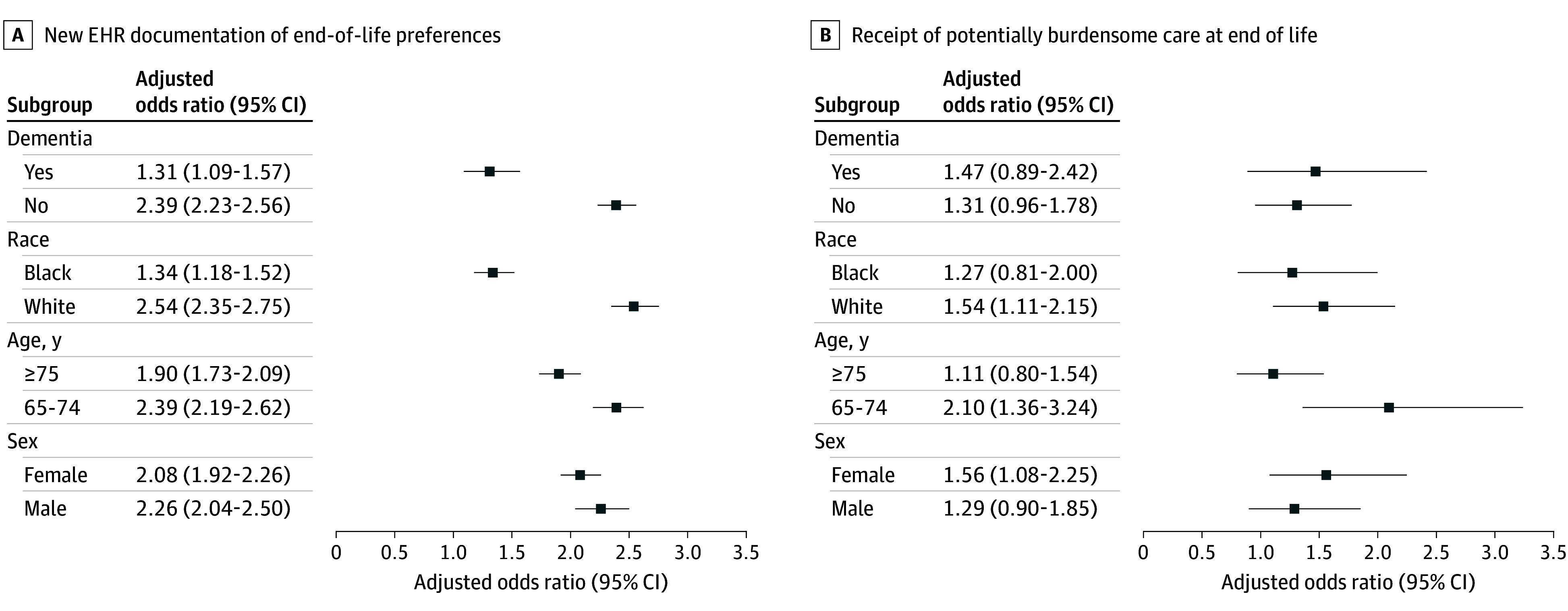
Adjusted Odds Ratios of the SHARING Choices Intervention vs Usual Care EHR indicates electronic health record.

The intervention effect for new and updated EHR-documented end-of-life preferences was significant in all subgroups (Figure 2; eTable 3 in Supplement 2). The effect was similar for both examined sexes but larger for older adults without diagnosed dementia compared with those with dementia, who were White compared with Black, and who were younger compared with older than 75 years. New documentation of end-of-life preferences was 3-fold higher for patients who engaged in ACP compared with those who did not (331 of 1031 [32.1%] vs 1859 of 17 283 [10.8%]) but varied 5-fold by practice (5 of 45 [11.1%] to 36 of 69 [52.2%]) (eFigure 2 in Supplement 2).

The percentage of decedents who experienced potentially burdensome care at end of life ranged from a low of 15.3% (90 of 590) among those with diagnosed dementia to 32.2% (139 of 432) among those who were Black (eTable 4 in Supplement 2). The intervention effect of greater potentially burdensome care at end of life was significant among intervention compared with control decedent subgroups by age, race, and sex (Figure 2; eTable 4 in Supplement 2).

## Discussion

This pragmatic cluster randomized clinical trial implemented ACP outreach and facilitation as a part of a multicomponent primary care intervention at 2 health systems. Less than 5% of intervention group patients engaged in facilitator-led ACP. The intervention increased new and updated documentation of end-of-life preferences among older primary care patients but also increased potentially burdensome care at end of life among decedents with serious illness. When assessed separately, intervention effects on new EHR documentation were larger for advance directives relative to a composite measure of end-of-life preferences. Importantly, effects for new and updated EHR-documented end-of-life preferences were higher for vulnerable subpopulations by older age, Black race, and dementia diagnosis.

End-of-life decision-making is complex, multifaceted, highly personal, and affected by individual, family, and setting-specific factors. We propose several explanations for our finding that potentially burdensome care at end of life was more common among decedents with serious illness in the intervention group, a finding that aligns with key critiques of ACP.^42^ Practice-level outreach that emphasized documentation of end-of-life preferences for all older adults and the availability of ACP facilitation at intervention practices may have inhibited deep, longitudinal conversations about preferences and values between clinicians and patients with serious illness and subsequent actions, such as communicating with a surrogate decision-maker.^43^ These ramifications may have been heightened in the context of care delivery challenges that were experienced during the ongoing COVID-19 pandemic.^20,21^ We note that potentially burdensome care for serious illness is an important pragmatic measure but is measured retrospectively with diagnosis and procedure codes that may not reflect patient and family experiences or the appropriateness of care at a challenging time.^44,45^

The smaller magnitude of intervention effects on EHR documentation of advance directives and end-of-life preferences for vulnerable subgroups reflect complex interpersonal dynamics affecting patient-clinician communication regarding serious illness^8^ and speaks to the value of equity reporting in interventional research.^46,47^ Older Black adults are half as likely as older White adults to engage in ACP or complete an advance directive^6^ yet more often receive worse quality serious illness care.^9,36,48^ Within the broader context of structural racism and mistrust,^8,36^ the study included facilitator training in equity issues.^20^ Facilitator-led ACP conversations occurred as or more often with older Black adults compared with older White adults,^21^ but we were unable to assess quality of conversations due to the pragmatic nature of the trial.

In contrast to prior primary care–based ACP interventional research,^12,49^ the SHARING Choices trial included older adults with dementia in recognition of the special importance of proactive planning for future care needs in this subpopulation.^41,50,51^ Dementia-specific advance directives have been developed,^52^ but systems and workforce issues in primary care pose implementation challenges that inhibit scaling in this setting.^53,54,55,56^ SHARING Choices aligns with best practices principles for approaching ACP in the context of cognitive impairment^57^ by seeking to normalize ACP with structured processes to support autonomy and decision-making preferences. The smaller intervention effect for EHR documentation of end-of-life preferences among patients with dementia suggests that additional outreach or support may be necessary to address unique considerations and challenges in this subpopulation.^10,57,58^

Our results are consistent with a broader literature indicating mixed clinically relevant outcomes of ACP.^59,60^ ACP is considered an important element of high-quality serious illness care, which by nature is complex and multidimensional, requiring holistic systems-level integration and collaboration.^5,61^ Collectively, our study and others reinforce the need for greater conceptual clarity, improved measures,^5,42,44^ and sustained systems-level investments in technological infrastructure and clinical supports to deliver high-quality serious illness care, such as through learning health systems initiatives.^62,63^

### Strengths and Limitations

Several strengths and limitations merit comment. As critiques of ACP have noted,^42^ EHR documentation of end-of-life preferences, which was prioritized in this pragmatic trial, is often challenging to easily access or apply. Despite a relatively large sample, the analytic cohort of decedents was relatively small due to lower than anticipated mortality, the constrained observation period, and our inability to ascertain out-of-state deaths. The study was implemented at 2 health systems with different EHR vendors operating primary care practices in diverse geographies, located in 1 region of the US, which inhibits generalizability. Limitations associated with relying on EHRs for ascertainment of our outcome of documented end-of-life preferences include the potential of measurement error, misclassification, and the inability to overcome gaps in cross-institution information exchange. The study reflects a pragmatic design and delivery of intervention components, with more limited uptake of the family agenda-setting checklist and shared portal components, and important variation in receptivity and delivery of the intervention that this analysis was not equipped to untangle.^19,21^ Study outcomes do not capture individual treatment preferences, and we are unable to evaluate person-reported measures about care quality or communication.

## Conclusions

In this pragmatic cluster randomized clinical trial, a multicomponent primary care ACP intervention for all older adults increased EHR-documented end-of-life preferences and especially advance directives in the overall cohort and key subgroups by age, sex, race, and dementia diagnosis. However, potentially burdensome care at end of life for decedents with serious illness was also increased. Study findings underscore the importance of comprehensive support for those with serious illness and prioritizing patient-relevant outcomes in ACP interventional research.

## Notes

Supplement 1. Trial Protocol

Supplement 2. **eTable 1.** Construction of Analytic Cohort for Analysis of Potentially Burdensome Care at End of Life **eTable 2.** Potentially Burdensome End-of-Life Care in Patients With a Serious Illness 6 Months or Before Death by Treatment Group, Procedure, and Subgroup **eTable 3.** Documentation of New and Updated End-of-Life Preferences by Type and Subgroup **eTable 4.** Potentially Burdensome End-of-Life Care Among Older Decedents With Serious Illness, 6 to 18 Months After Study Entry **eFigure 1.** Proportion of Older Adults Who Engaged in ACP at 19 Intervention Primary Care Practices **eFigure 2.** New EHR-Documented End-of-Life Preferences by ACP at 19 Primary Care Practices

Supplement 3. **Group Information.** SHARING Choices Investigators

Supplement 4. Data Sharing Statement
