# Supplementary material for: Advance Care Planning, End-of-Life Preferences, and Burdensome Care: A Pragmatic Cluster Randomized Clinical Trial
Source: JAMA Intern Med. 2024 Dec 2;185(2):162–70. doi: 10.1001/jamainternmed.2024.6215 (PMC11612918; doi:10.1001/jamainternmed.2024.6215)
Supplement: Supplement 1. — Trial Protocol [file jamainternmed-e246215-s001.pdf]

MANUAL OF PROCEDURES

SHARING Choices

Improving Communication for Primary Care Patients

Multi-Principal Investigators:

Jennifer L. Wolff, PhD, Johns Hopkins Bloomberg School of Public Health
Sydney M. Dy, MD, Johns Hopkins Bloomberg School of Public Health

Supported by:

The National Institute on Aging Award
R33AG061882

Version 3.0
Draft: August 19, 2024

National Clinical Trial (NCT) Identifier:
04819191

TABLE OF CONTENTS

Page

TABLE OF CONTENTS ii

PRÉCIS v

Study Title v
Objectives v
Design and Outcomes v
Interventions and Duration vii
Sample Size and Population vii

1 STUDY OBJECTIVES 1

1.1 Primary Objective 1
1.2 Secondary Objectives 1

2 BACKGROUND AND RATIONALE 1

2.1 Background on Condition, Disease, or Other Primary Study Focus 1
2.2 Study Rationale 1

3 STUDY DESIGN 2

4 SELECTION AND ENROLLMENT OF PARTICIPANTS 4

4.1 Inclusion Criteria 4
4.2 Exclusion Criteria 4
4.3 Study Enrollment Procedures 4

5 STUDY INTERVENTIONS 5

5.1 Intervention, Administration, and Duration 5
5.2 Adherence Assessment 6

6 STUDY PROCEDURES 8

6.1 Schedule of Evaluations 8
6.2 Description of Evaluations 8
6.2.1 Randomization 8
6.2.2 Identification of Patients 9
6.2.3 Evaluations 10

6.2.4 Study Completion 10

7 SAFETY ASSESSMENTS 10

7.1 Specification of Safety Parameters 10

7.2 Methods and Timing for Assessing, Recording, and Analyzing Safety Parameters 11

7.3 Adverse Events and Serious Adverse Events 11

7.3.1 Reporting Procedures 11

7.3.2 Follow-up for Adverse Events 12

7.4 Safety Monitoring 12

8 INTERVENTION DISCONTINUATION 12

9 STATISTICAL CONSIDERATIONS 13

9.1 General Design Issues 13

9.2 Sample Size and Randomization 13

9.2.1 Treatment Assignment Procedures 15

9.3 Interim analyses and Stopping Rules 15

9.4 Outcomes 16

9.4.1 Primary outcome 16

9.4.2 Secondary outcomes (Note that this section refers to "Assessment of Implementation" as opposed to quantitative examination of secondary outcomes) 18

9.5 Data Analyses 20

10 DATA COLLECTION AND QUALITY ASSURANCE 22

10.1 Data Collection Forms 22

10.2 Data Management 22

10.3 Quality Assurance 23

10.3.1 Training 23

10.3.2 Quality Control Committee 24

10.3.3 Metrics 24

10.3.4 Protocol Deviations 25

10.3.5 Monitoring 25

11 PARTICIPANT RIGHTS AND CONFIDENTIALITY 25

11.1 Institutional Review Board (IRB) Review 25

11.2 Informed Consent Forms 25

11.3 Participant Confidentiality 25

11.4 Study Discontinuation 26

12 ETHICAL CONSIDERATIONS.......... 26

13 PUBLICATION OF RESEARCH FINDINGS.......... 26

15 REFERENCES.......... 27

PRÉCIS

Study Title

SHARING Choices: Improving Communication for Primary Care Patients

Objectives

This study will evaluate the effectiveness and implementation of SHARING Choices among primary care patients ages 65 years and over including those with mild to severe Alzheimer's Disease and Related Dementias (ADRD). We will evaluate uptake of SHARING Choices components by patients and family and study outcomes through secondary analysis of information collected in routine clinical care.

Our primary objective is to quantitatively examine the effects of SHARING Choices on new documentation of end-of-life preferences in the electronic health record and receipt of potentially burdensome care at end-of-life for those who die with serious illness. Secondary objectives are to assess implementation and contextual factors that may facilitate or impede dissemination and sustainability.

Design and Outcomes

This is a pragmatic randomized, controlled trial. Randomization will be at the practice level. All components of SHARING Choices are currently available in primary care but have not been routinized in these practices. Practices randomized to control will continue with usual care, while practices randomized to SHARING Choices will have the therapeutic components made available to established patients ages 65 and older, including outreach on the components and availability of certified advance care planning (ACP) facilitators.

The study outcomes will be collected through secondary analysis of data collected for non-research purposes. Primary outcomes will be extracted from the electronic health record (EHR) and analyzed at the practice level. Outcomes include rates of newly documented end-of-life preferences and potentially burdensome care within 6 months of death for patients who die with serious illness. We examine effects for our overall cohort and for subgroups by diagnosed ADRD, age (<>75 years), sex, and race.

Our initial plans did not propose the inclusion of the Maryland MOLST in documentation of end-of-life preferences as the completion of the Maryland MOLST is mandatory in certain situations (e.g., transfer between settings of care) and is not indicative of ACP; it also does not conform to the National POLST Paradigm.[78,79] However, the Maryland MOLST and DC MOST have been identified as high priorities for our organizational partners, based on the relevance of these documents to care in the hospital. For this reason, we elected to operationalize our primary outcome of documentation of end-of-life preferences as a composite measure of a documented advance directive (durable power of attorney and/or living will) and/or the MOLST/MOST in the electronic health record.

Interventions and Duration

Primary care practices randomized to the control group will receive usual care, comprising standard primary care at participating practices. Primary care practices randomized to the intervention group will receive usual care in addition to SHARING Choices.

The SHARING Choices components include:

1. An invitation from the primary care practice introducing a new initiative,

2. Access to a facilitator trained in the protocol and leading ACP for persons age 65 and older including those with and without a diagnosis of ADRD,

3. A person-family agenda-setting checklist to align perspectives about the role of family and stimulate conversation about health care issues and ACP,

4. Information about the patient portal and how to register for it, to enable and extend electronic interactions and information access to patients and family, and

5. Implementation support, education, and resources about ADRD for practice staff.

Patients and families at SHARING Choices practices interested in ACP will have access to an ACP facilitator to schedule a conversation. SHARING Choices will be available to all patients 65 or older in these practices. Facilitators will be trained in all elements of the SHARING Choices protocol and will receive supplemental training to prepare them to lead ACP conversations for those living with ADRD. The overall duration of the study will be 18 months as measured from the date the first patients at intervention clinics are provided with information describing the SHARING Choices initiative.

Sample Size and Population

We will engage a diverse mix of primary care practices by geographic region (urban, suburban, rural) and size. The unit of randomization for this study is primary care practice. Study participants include all primary care patients ages 65 and over, including those with ADRD and their family at 51 primary care practices. Participating primary care clinics will have 2+ clinicians who care for patients ages 65 and older who collectively have established patient panels that include more than 500 older adults. We anticipate that 9,200 of 87,000 of older patients will have a diagnosis of ADRD, and 7,000 will die within 12 months of study entry.

1 STUDY OBJECTIVES

1.1 Primary Objective

This study will quantitatively examine the effects of SHARING Choices on new electronic health record documentation of end-of-life preferences and receipt of potentially burdensome care within 6 months of death for those with serious illness.

1.2 Secondary Objectives

Our secondary objectives are to assess effects on patient and family end-of-life experiences. We will assess contextual factors that may facilitate or impede dissemination and sustainability.

2 BACKGROUND AND RATIONALE

2.1 Background on Condition, Disease, or Other Primary Study Focus

Alzheimer's Disease and Related Dementias (ADRD) are among the most profoundly disabling and costly of all health conditions[1] and the 5th leading cause of death.[2] Family and friends (hereafter referred to as family) are at the forefront of managing ADRD across the continuum of care. Clinicians rely on the substituted judgement of family for persons who lack decisional capacity toward the end of life.[3-5] However, family members are not routinely engaged in conversations about prognosis[6,7] and are often poorly prepared for surrogate decision-making.[5,8,9] Compared to persons without ADRD, persons living with ADRD are less likely to complete an advance directive or formally designate a surrogate decision-maker,[10] placing them at heightened risk for unnecessary suffering and high utilization of burdensome and costly end-of-life care.[7,11,12]

2.2 Study Rationale

Engaging family in primary care is particularly important in ADRD because of the important role assumed in medical decision-making,[13-16] especially at the end of life.[17-19] Almost all (98%) community-living older adults with ADRD rely on the help of family caregivers.[20,21] Family caregivers of persons with ADRD provide hands-on care, coordinate information and care, and serve as surrogate decision-makers when persons with ADRD can no longer make decisions themselves.[20-22] Family caregivers of persons with ADRD actively participate in primary care visits,[23-25] and may inhibit or facilitate ACP.[19,26,27] Barriers related to lack of knowledge, fear, and reluctance are remediable with support and education.[12,28] A meta-analysis of 12 ACP interventions in community settings reported a statistically significant pooled effect size of 1.92 for advance directive documentation at 1-18 months follow-up.[28] Caregiving burden, stigma, and impaired decisional capacity amplify the difficulty and importance of ACP conversations for older adults with ADRD.[19,29] The few ACP interventions that specifically address the needs of persons with ADRD have been undertaken in nursing homes.[28,30,31]

Our study seeks to improve communication for persons with ADRD by establishing a structured protocol to proactively engage family in ongoing interactions with primary care and stimulate and support ACP and attention to ADRD in primary care throughout

the disease trajectory. Our premise is that individuals and families expect primary care practices to initiate ACP[32] and provide information and referrals for ADRD needs, [33,34] but that individual, family, and system factors including time, knowledge, and resources inhibit these conversations from occurring.[12] SHARING Choices seeks to better equip patients and family with the knowledge, skills, and support to engage in effective communication and ACP.

3 STUDY DESIGN

The study includes two phases that broadly correspond to Stages I (R61) and IV (R33) of the NIH Stage Model of intervention development.[25] In the R61 phase we refine the SHARING Choices protocol for delivery in primary care with input from primary care stakeholders (patients, family, clinicians and staff, health system administrators) and sought to strengthen relationships with health system collaborators (Aim 1). We further ensure feasibility and acceptability of delivering and evaluating the protocol through pilot-testing the protocol at our two partner health systems (Aim 2).

In the R33 phase we implement and evaluate the effectiveness of SHARING Choices on electronic health record documentation of end-of-life preferences and potentially burdensome care at end of life by conducting a cluster randomized pragmatic trial in 51 diverse primary care clinics from our 2 partner health systems (Aims 3 and 4). As implementation science stipulates the importance of considering dissemination and spread from the outset, we use a mixed-methods evaluation to qualitatively examine not only "if" the intervention is effective but also "how" and "why" it was successful and to identify facilitators and barriers of implementation that could affect subsequent dissemination (Aim 5).

This is a cluster randomized pragmatic trial that is embedded in primary care clinics. We test the effects of SHARING Choices on ACP outcomes for all older patients and persons with ADRD diagnoses in their electronic health record (EHR) (Aim 3). We plan a parallel mixed-methods design with a dominant quantitative strand (Aim 4) to examine effectiveness outcomes and a qualitative strand (Aim 5) to examine implementation and stakeholder perspectives. The R33 Phase seeks to develop the evidence to inform decision-making regarding subsequent adoption by primary care decision-makers. Therefore, the control protocol is usual care, or existing primary care practice.

Before randomization we assess each primary care clinic on panel size and composition (e.g., percent ages 65+, percent African American, number of clinicians, and geography). Patients from intervention clinics will receive the SHARING Choices protocol and patients from control clinics will receive usual care. Primary outcomes of new electronic health record documentation of end-of-life preferences and potentially burdensome care within 6 months of death for patients with serious illness or conditions will be assessed using information collected during routine care. We evaluate intervention uptake by patients and families overall and those with ADRD. After the trial we conduct key informant interviews and focus groups to assess diverse stakeholder perspectives on SHARING Choices and to identify facilitators and barriers that may affect sustainability and dissemination.

Components of SHARING Choices. The intervention comprises 5 components (See Exhibit 1):

1) an invitation from the primary care clinic introducing SHARING Choices to prepare patients and families to engage in ACP conversations,[35]

2) access to a facilitator trained in the protocol and leading ACP for persons with ADRD and their families,[36,37]

3) person-family agenda-setting to align perspectives about the role of family and stimulate conversation about health care issues and ACP,[38]

4) information about registration for the patient portal to enable and extend electronic interactions and information access to patients *and* family,[39,40] and

5) ADRD educational materials and resources for staff and clinicians.

We rely on Respecting Choices http:// respecting-choices.org, an existing ACP program that includes: patient and family education materials, a structured educational curriculum to train non-physicians in the competencies of ACP and standardization of policies for embedding ACP in routine care delivery,[36,37] and training specific to ACP in African-Americans, a subgroup expected to comprise 1/3 of our study population.[41] The Respecting Choices program includes 6 online modules with scripted interview tools and communication techniques to facilitate understanding ACP, exploring personal values, identifying a health care decision-maker, and communicating preferences for end-of-life care.

Exhibit 1. SHARING Choices Components, Content, Rationale, Evidence of Effectiveness

Content	Rationale	Evidence of Effectiveness
1. Primary care initiated voluntary ACP	Most patients expect primary care practices to initiate ACP.[32] Proactively introducing ACP normalizes these conversations.	Primary care initiatives to increase advance directive documentation are effective and well-received.[35,49]
2. ACP education and availability of non-clinician led ACP conversations	ACP videos increase patient & family awareness, knowledge and skill. [17,18] Respecting Choices is a structured educational program to train facilitators to facilitate ACP conversations.[42,43]	ACP is associated with delivery of goal concordant care, greater confidence among surrogate decision-makers[3,11,50,51] & reduced EOL costs.[36,37,42,43]
3. Person-Family Agenda Setting	Individuals & families often have different concerns. Agenda-setting stimulates conversations about ACP & the role of family.	Agenda-setting helps clarify concerns, goals, and expectations, and increase engagement in care.[38,52,53]
4. Access to Electronic Health Record Via Patient Portal	The patient portal facilitates timely and accurate information about patient health, diagnoses, test results, & prescribed treatments. Families are provided their own identity credentials to access information and communicate with clinicians.	The patient portal operates through mechanisms of convenience, continuity, activation, and understanding.[54] Prior studies find clinical benefit of supporting family through technology.[55-59 40]
5. ADRD Resources and Training of Staff and Clinicians	Patients and family expect primary care practices to provide reliable ADRD information [33,34] but	Tool kits,[61] training curriculum,[62] billing codes,[63] & referral resources[64] support ADRD-friendly

	practices are often unprepared.[60]	primary care practice.
**ACP=advance care planning; EOL=end-of-life; PCP=primary care practice**		

4 SELECTION AND ENROLLMENT OF PARTICIPANTS

We conduct the study in partnership with two primary care clinic health systems which together operate more than 75 primary care clinics in the Baltimore-Washington DC metropolitan corridor. Both systems have integrated electronic health records (EHRs) with consumer-facing patient portals and innovative care models such as the Maryland Primary Care Initiative. One health system operates a centralized palliative care program. Participating primary care clinics will have 2+ clinicians whose panels include older adults that collectively include more than 500 older adults.

In this pragmatic trial we target all primary care patients ages 65 and over, including those with and without ADRD. There is no formal enrollment of participants into this pragmatic trial as this is a clinic-level initiative that will be available and offered to all eligible patients at clinics randomized to the intervention. We expect 9,200 of 87,000 patients ages 65+ will have a diagnosis of ADRD as further defined in section 9.5, and 7,000 (8%) will die within 12 months of study entry.

4.1 Inclusion Criteria

Practices:

- Affiliated with one of our health system partners, and
- Primary care practice, defined as adult internal medicine, family medicine, and geriatric medicine.
- 2+ practicing clinicians
- More than 500 patients ages 65 and older receiving care at the practice

Patients:

- Age 65 and older, and
- Established patient of primary care clinician at participating practice (>1 prior visit at the clinic)

4.2 Exclusion Criteria

Practices:

- Fewer than 2 clinicians.
- Fewer than 500 established patients ages 65 and older
- Not a part of a partnering health system.

Patients:

- Less than 65 years of age

4.3 Study Enrollment Procedures

N/A

5 STUDY INTERVENTIONS

5.1 Intervention, Administration, and Duration

Patients of primary care clinicians at practices randomized to the ***control*** group receive a protocol of usual care, comprising standard primary care at participating practices. Patients of primary care clinicians at practices randomized to the ***intervention*** group receive usual care in addition to SHARING Choices components (see Exhibit 1 and Supplemental Appendix I) including:

1) An electronic notice or letter from the primary care practice introducing SHARING Choices, including ACP,
2) A person-family agenda-setting checklist to align patient and family perspectives regarding the role of the family member in primary care interactions and stimulate interest in ACP,
3) Access to a trained and certified facilitator to lead ACP conversations, and distribution of AD forms,
4) Information about the patient portal and how to register for it, to enable and extend electronic interactions and information access to patients and family, and
5) Implementation support, education and resources about ADRD for practice staff, tailored to SHARING Choices practices.

To maximize scalability, SHARING Choices is designed for compatibility with team-based care, minimally disruptive to primary care workflows, and standardized yet adaptable. Delivery characteristics vary by organizational partner and clinic due to the intrinsic variability in how clinics are staffed and information is managed. Patients and families at intervention practices interested in ACP have access to a trained ACP facilitator to schedule a conversation. All facilitators are trained in all elements of the SHARING Choices protocol, with supplemental training for ADRD.

ACP conversations include an initial meeting that is flexible in location and modality. These meetings may occur by phone or through telehealth, and at the primary care clinic, in the community, or at the individual or family residence. In the initial meeting the facilitator follows the Respecting Choices structured conversation guide and employs motivational interviewing techniques to evaluate readiness and facilitate discussion of SHARING Choices components. During ACP conversations, facilitators review the program components with patients and family. The duration and nature of the conversations and resulting decisions tailored to individual preferences are recorded for each patient meeting in the electronic medical record operated by our two health system partners. ACP conversations may be delivered at multiple time points through patient and family requests and clinician-referrals. We expect that ACP conversations will generally be at least 15 minutes in duration. The overall duration of the study will be 18 months as measured from the date the first patients at intervention clinics are provided with information describing the SHARING Choices initiative.

Handling of Study Interventions

The study team embedded the interventions in care using a phased approach. **First**,

investigators identified liaisons from each of the two partnering health systems to assist with the implementation of the intervention at participating clinics. Upon receipt of our notice of award for the R33 trial, these liaisons to the study worked with their health systems' organizational leadership to identify and contact office medical directors and administrators to introduce the initiative to primary care clinics that have been randomized to the intervention.

Second, the study team scheduled times to discuss SHARING Choices with the office medical directors and administrators of primary care clinics that were randomized to the intervention. The study team worked with the medical director and administrator to meet with staff and clinicians at monthly meetings at clinics that were randomized to SHARING Choices.

- *Clinic staff* have been introduced to the initiative at monthly staff meetings, followed by multimodal training (as appropriate) for key clinic staff members including at least one front office or clinical support staff.
- *Front desk staff* have been informed about the study objectives and how to respond to patient/family queries that may arise related to intervention processes such as how to facilitate registration to the patient portal for families, and where and how to upload a completed advance directive. *Clinicians* have been informed of an opportunity to refer patients to the clinic's trained ACP facilitator following the documentation of an annual wellness visit questionnaire, preceding or following a hospitalization, or during other transitions of care. This training has emphasized the opportunity to refer patients to the ACP facilitator. The description of the protocol for training of ACP facilitators is described in 10.3.
- *Patients* and *families* from intervention clinics are learning about the initiative in several ways, including by clinician or staff referral, through brochures, through mailed letters, and through patient portal outreach via secure or text messaging to eligible patients (See 6.2.3, Identification of Patient Participants). Patients are being provided with contact information for the clinic's ACP facilitator via mailed letter and through informational study materials posted or otherwise made available through intervention clinics. Patients are being invited to complete or utilize mailed materials, and to contact the clinic ACP facilitator as desired.

As a pragmatic trial in which intervention processes are integrated into routine care, there is no blinding of the study team or clinicians and staff at primary care clinics. Interactions with the study team, the training of clinic staff and clinicians, and the mailing of letters to patients ages 65 and older clearly identify which clinics have been randomized to SHARING Choices or usual care.

5.2 Adherence Assessment

Adherence assessment refers to monitoring and maximizing fidelity to the intervention protocol. Guided by the NIH Behavior Change Consortium[65] we address fidelity through a.) design, by selecting distinct therapeutic elements that are based on theory, b.) training, by relying on a protocolized curriculum to train facilitators in advance care planning and other elements of SHARING Choices, c.) monitoring uptake of intervention processes, and d.) ongoing training support and resources to support facilitators and confirm fidelity to intervention procedures.

As a pragmatic trial, tracking of intervention processes are monitored through data collected in routine interactions. Data monitored via the electronic health record include documented ACP conversations, completed advance directives and patients and proxy/care partner registration and use of the patient portal. Additionally, the study team collects limited information from ACP facilitators related to safety monitoring (See 7.3).

Post-ACP Meeting Documentation: ACP facilitators document conversations using structured fields in the electronic health record. After each ACP conversation, facilitators document their impressions of meeting content and progress, including the structure of the meeting (individuals present, location, meeting duration), whether an advance directive was completed, and whether a health care agent was named. ACP facilitators at Health System 2 document outcomes of interactions with patients using the Cerner electronic health record using workflows and PowerForms. ACP facilitators at Health System 1 report outcomes of interactions with patients in EPIC using standard workflows and a customized visit note template.

Ongoing Supervision for Facilitators: ACP facilitators are regularly convened prior to and throughout the trial. The content and periodicity of meetings vary based on study phase. Prior to the launch of the trial, the primary focus was to educate facilitators about the study objectives, workflows, and protocols and to build skills in ACP. After the trial launches, facilitators are provided study updates and asked to present cases, troubleshoot challenges, engage in collaborative problem-solving, and discuss strategies for resolution. The structure and content of meetings are tailored to be responsive to facilitators' specific needs. Supervision meetings are used to administer booster sessions to maintain skill, remediation in response to identified challenges, and to minimize drift in fidelity to the Respecting Choices conversation guides. Ad-hoc support is available to individual facilitators as needed. Just-in-time supervision was available for all ACP Facilitators for any issues or guidance. At Health System 1, the Research Program Manager responded to Facilitator issues and responded with solutions related to clinical workflows, staffing, or record-keeping. The site PI at Health System 2 was available during business hours to resolve issues related to EHR functionality, patient interaction, and clinic embedding. A simulated exercise was required for all Facilitators at the midway point of the trial duration. The exercise included an observed ACP interaction with simulated patients scored based on a rubric designed to overlap with the Respecting Choices conversation guide. Following the exercise, participants were offered feedback and tailored training.

Field Observations: Ongoing supervision for facilitators (noted above) was supported through regular contact with facilitators at both participating health systems through probing for feedback regarding practice-level systems and workflows that are working well or that pose barriers to maintaining fidelity to study processes. This insight is used to trigger consideration regarding the need to adapt workflows and processes.

Progress Reports: Both participating health systems have system-wide dashboards to track quality improvement initiatives. Advance directive documentation is an active area of quality improvement at both systems due to its inclusion in the Maryland Primary Care Initiative. These dashboards are used to identify clinic-level documentation of advance directives and MOLST/MOSTs. At the clinic level, these Dashboards are used by the study team to monitor progress real-time and enable early corrective action to

remedy identified challenges. Facilitators use these Dashboards to identify patients with upcoming appointments who are eligible and have not yet had an advance care planning conversation. As a clinic-level initiative the study team has prioritized working with each intervention clinic to identify clinic-specific targets at the inception of the study and will track progress toward meeting clinic-level identified goals. At the conclusion of the study, information from the Dashboards will be available to descriptively characterize clinic-level differences in advance directives/MOLST/MOST documentation and assess how implementation relates to observed end of life outcomes.

6 STUDY PROCEDURES

6.1 Schedule of Evaluations

The schedule of evaluations is presented in Table 6.1. Before randomization we assess each primary care clinic on panel size and composition (e.g., age, race, payer mix), clinician/staff composition (numbers, types of discipline), and percentage of patients with documented advance directives. Patients from intervention clinics receive the SHARING Choices protocol and patients from control clinics will receive usual care. Primary outcomes of electronic health record documentation of end-of-life preferences and potentially burdensome care at the end of life are assessed using information collected during routine care.

Assessment (Data Source)	Clinic Randomization (Day -180)	Baseline: (Day-45 to Day -1)	ACP, Patient Portal Use	12 Months Day 365 (±30 Days)	EOL (±30 Days)
Randomization of Eligible Primary Care Practices (Health System Data)	X				
Identification of Patient Participants (Health System Data)		X			
Delivery of Intervention (ACP Facilitators, Health System EHR Data)			X		
Outcome: New Electronic Health Record Documentation of End of-Life Preferences (Health System EHR Data)				X	
Outcome: Potentially burdensome Care at EOL (Health System EHR Data; CRISP Pull)					X
Adverse Events			X		

6.2 Description of Evaluations

6.2.1 Randomization

The unit of randomization is the primary care practice, which avoids the potential for contamination of controls, affords efficiency in staff training, and enables practice-level deployment. We first stratify practices by health system as variation in system-level processes, culture, and commitment to the intervention could affect outcomes. Second, we balance intervention and control groups on practice characteristics:

geography (urban, suburban, rural location), percent of patients of African American race, percent of patients ages $\geq$ 65, number of clinicians. An initial randomization protocol was conducted by the statistician in April 2020, preceding the launch of the trial. The PIs, study team, and our organizational health system partners were blinded to clinic randomization until the inception of activities relating to the launch of the trial in September 2020.

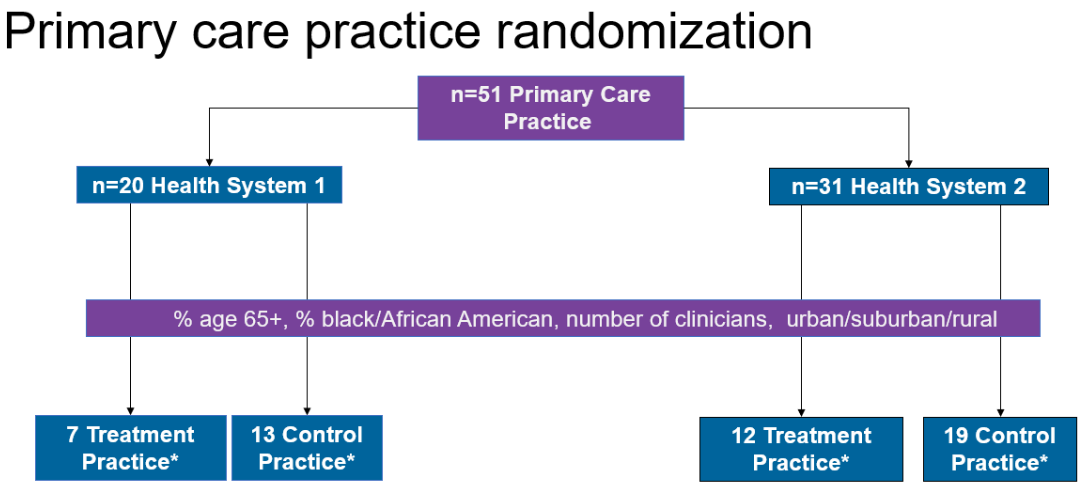

*A covariate-constrained cluster randomization approach was used to optimize balance on key site characteristics (*Huang & Roth, 2021, Trials*). We assigned practices in a **1:2 ratio** because control practices incur no costs to the study and having more control practices affords greater precision in outcome estimates.

6.2.2 Identification of Patients

Although both health system partners use a centralized approach to identify patients, the specific systems and workflows differ based on variability in electronic medical records and supporting information systems. The study team developed standing data requests to centrally identify candidate patients who meet eligibility criteria from the electronic health records of each of the participating practices (both intervention and control) from both health systems. At Health System 1, this report is produced twice monthly and stored on the SAFE Desktop secure servers. The report is used to centrally generate mail-merge processes to patients under the care of clinicians at intervention practices who have scheduled upcoming visits within 4 weeks. At Health System 2, a similar process is followed whereby eligible patients from both intervention and control practices are identified weekly using data from IDX/Cerner. Eligible patients include any patient with an appointment scheduled two weeks out from the date of the data pull. Weekly data extracts are uploaded to a REDCap database within the firewall to prospectively build the analytic sample. Weekly reports using the Discern reporting portal platform are shared with the mailing team that will create mailing labels for patients in the intervention group. Eligible patients due for appointments at intervention practices will also be included in a MPage report accessible to the ACP facilitators from Health System 2.

Onboarding meetings are held with practices randomized to implement SHARING Choices to review the purpose of the program and implementation. Through ongoing meetings with practice champions, medical directors, staff, and administrators, each health system has identified a change team to design and

document the local implementation strategy. At both participating health systems, patients who receive care at intervention practices are provided information describing SHARING Choices and an invitation to make use of available services. The study team tracks and retains dates of upcoming visits that are used to trigger the mailing of invitations to learn about the SHARING Choices program. The initial visit date for each candidate patient after the inception of the trial serves as the beginning of the 12-month observation period at intervention and control practices. We use this initial visit date to construct comparable 12-month observation periods for candidate patients at both intervention and control groups across both partner systems.

6.2.3 Evaluations

Our primary outcome of documentation of end-of-life preferences is defined as a durable power of attorney, living will, or Maryland MOLST (or DC MOST) included in the electronic health record at 12 months (see 9.4.1). Our primary outcome of potentially burdensome care is defined as any aggressive procedures (intubation and mechanical ventilation, tracheostomy, gastrostomy feeding tube placement, hemodialysis, and enteral and parenteral nutrition) within 6 months of death for patients with serious illness or conditions using dates and services extracted from our regional health information exchange, the Chesapeake Regional Information System for our Patients (CRISP; see 9.4.1). Adverse events are defined in our Data and Safety Monitoring Plan. Adverse events will be monitored on an ongoing basis by study staff using a tracking form (see 7.3.1).

6.2.4 Study Completion

Study completion is defined on the basis of duration from the time that the intervention practice "goes live" as defined by the study team from the date of initial mailed letters to eligible patients of clinicians from sites randomized to the intervention group.

7 <u>SAFETY ASSESSMENTS</u>

Participant safety will be monitored as follows. ACP facilitators will be asked to identify and report Adverse Events and Safety Alerts. Definitions of events and a description of safety assessments and reporting is further described in the following text.

7.1 Specification of Safety Parameters

This is a minimal risk trial of a behavioral intervention involving ACP. Risks of participation are no more than encountered in routine clinical care: elements of the intervention are already widely available in routine care. As such, reporting on safety parameters related to serious adverse events is not applicable. Facilitators will report potential emergency situations involving unanticipated events that arise during an ACP meeting which require immediate action such as evidence of abuse, suicidal ideation with intent, or emergent medical emergencies.

It is possible that some persons and family members who choose to have an ACP conversation with the ACP facilitator may become upset or experience discomfort by responding to questions about wishes for future medical care, by participating in agenda setting or ACP conversations. ACP is an accepted standard in clinical care where

benefits outweigh risks, and we will institute several accepted mechanisms to reduce the potential for psychological discomfort. SHARING Choices facilitators will receive training in techniques for approaching persons, including those with ADRD and family members, about ACP. Additional training in sensitivity to discussing topics related to ACP and cultural competency for working with diverse populations where there may be specific needs for these conversations will be required.

7.2 Methods and Timing for Assessing, Recording, and Analyzing Safety Parameters

See 7.1; not applicable.

7.3 Adverse Events and Serious Adverse Events

A trained ACP facilitator will be available to discuss questions or concerns about ACP. If concerns that are distressing come up in the course of facilitation conversations, ACP facilitators will follow normal practice workflows for assessing patient safety concerns including involving a triage nurse or provide this information to primary care providers so that appropriate clinical actions can be taken. As with all standard care, patients and families may decide to stop ACP facilitation at any time.

As with all standard care, ACP is voluntary for patients, and ACP sessions will only be initiated or terminated at the direction of the patient. While it is possible that agenda setting or ACP may introduce tension in the person-family relationship by acknowledging the distinct perspective of each individual, extensive research has also found significant benefits with these conversations, and facilitators will be trained to anticipate concerns and mediate difficult conversations.

Family of older persons with significant health conditions, including those with Alzheimer’s Disease and Related Dementias, are at heightened risk of depression and anxiety. Should this come up as in issue, the ACP facilitator will ask the family if they would like an educational brochure about depression or anxiety (as appropriate) and encourage them to follow up to discuss this with their clinician. In the event of extreme psychological discomfort among patients or families in ACP conversations, ACP facilitators will defer to the practice’s guidance on whether and how to make necessary medical or professional referrals.

Occurrences fitting the definitions for Adverse Events and Safety Alerts will be documented and categorized by facilitators.

7.3.1 Reporting Procedures

As this is a pragmatic trial in which the intervention is woven into the practice’s daily practice and is carried out by trained facilitators, reporting of Adverse Events and Safety Alerts will be monitored. Facilitators will document adverse events for review by program leadership. The research team will confer with the health system partners and/or medical office directors prior to the initiation of the trial to come to a consensus as to whether additional reporting procedures are necessary for this pragmatic trial. Concerns unlikely to have resulted from the intervention will be summarized for routine DSMB reporting in a separate table prepared by the study biostatistician.

7.3.2 Follow-up for Adverse Events

The occurrence of an adverse event will only come to the attention of study personnel via report from ACP facilitators. A list of adverse events reported during the trial will be maintained by the study team using the adverse event form. Information to be collected includes event description, date identified, facilitator assessment of severity, response actions taken, and the relationship to the trial intervention. All adverse events that are reported to the team will be documented regardless of relationship to the intervention. Where possible through facilitator team huddles or case reports, outcome information, response, or resolution will be recorded.

Adverse events will be reported per IRB policies. All members of the DSMB will receive copies of all safety reports at the time of submission to the IRB of the Johns Hopkins University. In addition, a listing of all adverse events and their attribution (e.g., study related, intervention related, or unrelated to study or treatment) will be provided to the DSMB. Should an adverse event occur that is unanticipated and related to the intervention, it will be reported to NIA Program Officer and to the DSMB Chair or to the designated DSMB member within 48 hours of the study's knowledge of the adverse event. All other significant adverse events will be reported to the NIA Program Officer and to the DSMB (or a Safety Officer) quarterly.

7.4 Safety Monitoring

The Data and Safety Monitoring Board (DSMB) will act in an advisory capacity to the National Institute of Aging (NIA) Director to monitor participant safety, data quality and evaluate the progress of the study. The DSMB will be responsible for reviewing the safety of study participants during the conduct of this trial and providing recommendations to the research team on specific aspects of the research protocol as it pertains to safety and adverse events. The DSMB Charter provide additional details regarding the trial approach to safety monitoring.

8 INTERVENTION DISCONTINUATION

This is a Minimal Risk study. The trial will be conducted in a population of older adults and the significance of our study rests in part on low documentation of advance care planning in the target population despite high rates of mortality and adverse health events (e.g., hospitalization, emergency department visits). Advance care planning is already routinely offered in primary care and uptake of intervention processes will be voluntary and the decision of patients and families. Participants in the trial will be offered the intervention as a part of routine care and will be free to decline all aspects of intervention activities but will continue to be analyzed according to intention to treat. For these reasons, we do not include plans for intervention discontinuation.

9 STATISTICAL CONSIDERATIONS

9.1 General Design Issues

This study is a cluster-randomized, parallel group trial with primary care practices stratified by health care system and patients nested within practices. The unit of randomization is the primary care practice, which avoids the potential for contamination of controls, affords efficiency in staff training, and facilitates the feasibility of applying the SHARING Choices intervention within the practice. The trial will be conducted at 51 diverse primary care practices from 2 health systems. We plan a parallel mixed-methods design with a dominant quantitative strand to examine effectiveness outcomes and a qualitative strand to examine the implementation processes and stakeholder perspectives of SHARING Choices. Our primary outcomes of end-of-life preferences documentation at 12 months and receipt of potentially burdensome care within 6 months of death will be extracted from electronic health records and the Maryland CRISP by study staff masked to treatment group. These measures were selected because having an advance directive is strongly associated with less aggressive, costly, and burdensome end-of-life care among persons with ADRD[66-68] yet persons living with (versus without) ADRD are less likely to participate in ACP, appoint surrogate decision-makers, or complete living wills.[10,66] As prior studies of ACP in primary care have enrolled modest numbers of persons and targeted a general population at low risk of mortality,[12,28] little is known about effects on end-of-life care.[69] Our study will therefore contribute to an evidence gap in quantifying the effects of advance care planning on end-of-life care.

9.2 Sample Size and Randomization

The unit of randomization is the primary care practice. Practices are embedded within health systems. Randomization is therefore first stratified by health system and balanced on practice characteristics, including percent of patients ages 65+, percent of patients that are African American, number of clinicians, and geographic location (urban, rural, suburban). Sample size estimates are based on the following considerations: (a) the cluster-randomized design of our trial, (b) ability to detect effect sizes comparable to other treatment effect estimates reported in the published literature,[28] and (c) two-tailed type 1 error rate of 0.025 due to having two primary outcomes in the trial. The two primary outcomes will be 1) completed documentation of end-of-life preferences in the electronic health record at 12 months and 2) receipt of potentially burdensome care within 6 months of death among patients who die with serious illness. We expect approximately 87,000 older adults from 55 primary care practices to be eligible, of whom approximately 9,200 will have a diagnosis of ADRD or cognitive impairment and 7,000 will die within 12 months of entry into the study (see Table below and Recruitment and Retention Plan).

Sample Size	Full Sample	Intervention	Control
All Patients Ages 65+	87,000	29,000	58,000
Cognitive impairment or ADRD	9,200	3,100	6,100
Mortality at 12 Months	7,000	2,400	4,600

Power calculations incorporate two features of SHARING CHOICES: 1) that practices will be randomized to intervention versus control at a 1:2 ratio, 2) that effects of clustering are taken into account by assuming variability in the control group success rate (analogous to an intraclass correlation assumption for a continuous outcome). Because primary outcomes are assessed using data collected in routine care as opposed to patient-reported information, attrition is expected to be low. All power calculations were performed using the Optimal Design software package for cluster randomized trials.[70,71]

For our primary outcome, **documentation of end-of-life preferences**, we initially planned to focus on advance directive documentation as our endpoint. Preliminary data indicates that advance directive documentation in the electronic health record is relatively uncommon and varies by practice across our two health system partners. If we assume advanced directive documentation of 15% at baseline (the base rate at Health System 1)[72] and a plausible range of ± 10% (i.e. from 5% to 25%) based on nationally-representative estimates, we have power of 0.80 to detect an increase from 15% to 19%. A graph of available power for the outcome of advance directive documentation is depicted in **Figure 1**. Because 29,000 patients are expected to be exposed to the intervention, an increase from 15% (4,350 persons) to 19% (5,510 persons) represents an increase in advance directive documentation among 1,160 older adults at intervention practices.

For the outcome of **potentially burdensome care**, we project that 7,000 of the 87,000 patients in the trial (8%) will die within 12 months of intervention exposure, based on nationally-representative mortality rates.[73] Based on data from the Health and Retirement Survey we assume a base rate of potentially burdensome care of 18%, with a plausible range of ± 5% (i.e., from 13% to 23%).[66] From these assumptions, we have power of 0.80 to detect a reduction in potentially burdensome care from 18% to 15% for the 7,000 patients who die. The available power for the outcome of potentially burdensome care is displayed graphically in **Figure 2**. Because 2,333 of these deaths are expected to occur in intervention practices, we have sufficient power to detect a reduction in receipt of potentially burdensome care from 18% (420 persons) to 15% (350 persons) in this group.

- **Figure 1**

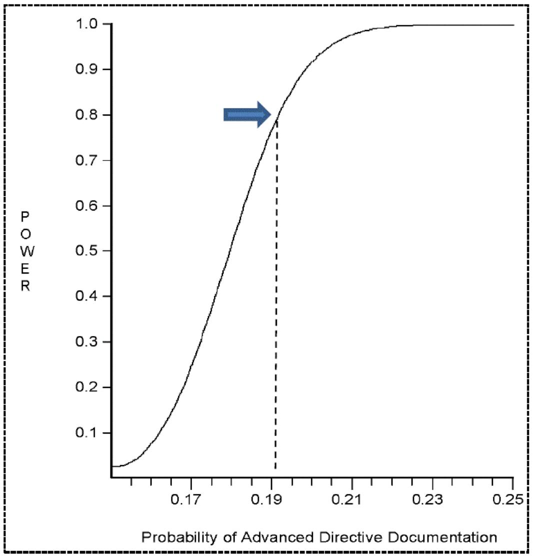

Figure 2

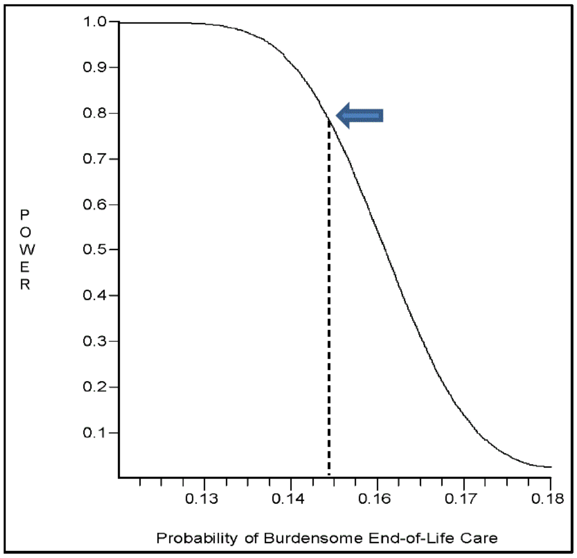

9.2.1 Treatment Assignment Procedures

The unit of randomization is the primary care practice, which avoids the potential for contamination of controls, affords efficiency in staff training, and enables practice-wide deployment. We first stratify practices by health system as variation in system-level processes, culture, and commitment to the intervention could affect outcomes. Second, we measure and balance intervention and control arms on practice characteristics (e.g., urban, suburban, rural location, patient mix by race, age $\geq$ 65). We assign practices to intervention or control arms in a 1:2 ratio because control practices incur no cost to the study and having more control practices will afford greater precision in estimates of study outcomes. Because we include 20 practices from Health System 1 and 35 practices from Health System 2, notable and problematic imbalances on practice-level characteristics may easily occur by chance in a single randomization. Methodological innovations are needed to achieve balance on multiple practice-level characteristics while still maintaining the experimental rigor and inferential value of true random assignment.

For this reason, we used a covariate-constrained randomization (CCR) method to assure approximate balance on selected practice characteristics across intervention and control groups. In this CCR, for each organization, 1,000 randomizations were undertaken using a 1:2 assignment ratio. A subset of randomizations that met 5 balancing characteristics (urban vs. suburban, rural vs. suburban, # of clinicians, % age 65+, and % Black) and the following 3 criteria were then identified:

1. The overall balance index, I, based on the half-normal distribution of the absolute value of Sharing Choices vs. Control differences on the balancing variables, was in the bottom 10%. That is, only the best 100 of the 1,000 randomizations were further considered, following Huang. [74]
2. The p-value from a non-parametric test, the Kruskal-Wallis (KW) test, had to be > 0.30 for each balancing variable. This is the method recommended by Ciolino et al. (2019) for use in CCRs. [75]
3. For Health System 1, 1-2 urban and 2-3 rural practices had to be assigned to SHARING Choices; for Health System 2, 3-4 urban and 2-3 rural practices had to be assigned to SHARING Choices.

For Health System 1, 79 of the 1,000 randomizations met all three criteria, and for Health System 2, 41 of the 1,000 randomizations met all three criteria. From the 79 Health System 1 and 41 Health System 2 eligible randomizations, 1 was randomly selected for each organization for the purposes of our preliminary randomization. Of the 55 practices for this study, 19 were randomized to SHARING Choices and the remaining 26 practices were randomized to the control group.

9.3 Interim analyses and Stopping Rules

This is a pragmatic trial of advance care planning, which is already available in routine care. As noted in 9.4, outcomes to be assessed require data extraction from health system electronic health records and our state health information exchange and will be evaluated at the conclusion of the trial. Therefore, we do not plan interim analyses, although the

DSMB will ultimately be responsible for devising stopping rules.

9.4 Outcomes

We selected measures based on: 1.) known reliability and validity, 2.) sensitivity to change, 3.) clinical relevance, 4.) reflection of objective indicators of the domains we seek to impact, and 5.) feasibility of data extraction within the context of the pragmatic trial funding mechanism. Measures are primarily extracted from the EHR and Maryland-DC health information exchange, CRISP. At the completion of the study, staff masked to treatment group will extract measures of documentation of end-of-life preferences from the EHR and potentially burdensome care from CRISP. We additionally will assess implementation of the intervention and its delivery context.

9.4.1 Primary outcome

Electronic Health Record Documentation of End of Life Preferences is defined as a durable power of attorney, living will, Maryland Medical Order for Life Sustaining Treatment (MOLST), or District of Columbia Medical Order for Sustaining Treatment (MOST; see Box) based on information that is recorded in each care delivery system's electronic medical record 12 months after study entry.[18,76-79] To operationalize this outcome, we will separately examine advance directive and MOLST documentation among patients in the target populations of interest at intervention and control practices. Having an advance directive is strongly associated with less aggressive, costly, and burdensome end-of-life care persons with ADRD.[66-68] However, the completion of an advance directive alone has little influence on end-of-life decisions in the absence of informed, thoughtful reflection of individual wishes and values, and personal communication between an individual and their likely decision-maker. [66 80-82]

Our initial grant proposal did not propose the inclusion of the Maryland MOLST as the completion of the Maryland MOLST is mandatory in certain situations (e.g., transfer between settings of care) and is not indicative of ACP; it also does not conform to the National POLST Paradigm.[78,79] However, the Maryland MOLST and DC MOST have been identified as high priorities for our organizational partners, based on the relevance of these documents to care in the hospital. For this reason, we have elected to operationalize our primary outcome as a composite measure of a documented advance directive (durable power of attorney and/or living will) and/or the MOLST/MOST in the electronic health record. This approach has been used in other pragmatic health system-based trials of advance care planning.[83]

Although our primary outcome will be operationalized as a composite measure, we examine each component outcome in parallel for older adults ages 65 and older with and without a diagnosis of dementia as defined by ICD-10 diagnosis codes that are noted in the table located in 9.5. By separately examining between-group differences in advance directive and MOLST/MOST completion for older adults with and without a diagnosis of dementia we will be able to assess whether the study differentially affected these two very different outcomes for the relevant subgroups.

**Box**: Definitions Relevant to Primary Outcome (See Bomba; 2012) [84]
**Advance Directive**: is a statement of wishes regarding future medical treatment options that are intended to be completed by all adults. There are typically two components: 1.) a **durable power of attorney** designating a health care agent who will make decisions should an individual lose the ability to make choices, and 2) a **living will** which is a legal document that details personal choices about receiving specific end-of-life treatments and procedures
**Physician Orders for Life Sustaining Treatment** (POLST https://polst.org/about-national-polst/?pro=1): is a national term referring to portable medical orders intended for patients who are considered to be at risk for a life-threatening clinical event because they have a serious life-limiting medical condition or are frail. POLST is completed by a health care professional and unlike advance directives may be completed through discussions with a health care agent for patients who lack capacity. POLST has different names in different states – most relevant to this study is the Maryland MOLST https://marylandmolst.org/ and DC MOST https://dchealth.dc.gov/most.

Potentially burdensome care at end-of-life will be measured as any (yes/no) aggressive procedures within the 6 months that precede death using dates and validated ICD-10 codes for hospital services that will be extracted from CRISP, the regional health information exchange, which includes a repository of all hospital encounters in Maryland, Delaware, West Virginia, and the District of Columbia.[66,68,85] Specific procedures and codes that will be used to reflect potentially burdensome care include intubation and mechanical ventilation, tracheostomy, gastrostomy feeding tube placement, hemodialysis, enteral and parenteral nutrition, and cardiopulmonary resuscitation. The specific procedures and codes to construct this measure originate from the Dartmouth Atlas as reported by Barnato (2009) and are presented in the table below. The denominator for our measure of potentially burdensome care will comprise patients who experienced a death during the observation period from dates of death that are recorded in the electronic health record at each of our health system partners and CRISP, the Maryland regional health information exchange. We will limit the denominator for this measure to patients with serious illness conditions at the time of death for whom these procedures would be considered potentially burdensome, drawing from a modified list of ICD-10 codes reported in Walling et al, (2019), listed in the table below.

Codes for Potentially Burdensome Care at the End of Life (Numerator)

Procedure	CPT/HCPCS Code	ICD-10
Intubation and Mechanical Ventilation	31500	OBH17EZ, OBH18EZ, OB717DZ, OB718DZ, 0BH07DZ, OBH07YZ, OBH172Z, OBH17YZ, OBH182Z, OBH18YZ, OBHK7YZ, OBHK8YZ, OBHL7YZ, OBHL7YZ, OBHL8YZ, OWHQ7YZ, 5A19, 5A1935Z, 5A1945Z, 5A1955Z, 5A12012, 5A19054
Tracheostomy	31600, 31601, 31603	OB11, OB110F4, OB11OZ4, OB113F4, OB113Z4, OB114F4, OB114Z4,
Gastrostomy Tube Insertion	49440, 43761	
Hemodialysis	90935, 90937	5A1D, 5A1D00Z, 5A1D60Z
Enteral or Parenteral Nutrition	43750, 43246, 44372, 44373, 74350, 43832, 43830, 43653, 49440, 49441, 49446	0DH67UZ, 0DH68UZ, 3E0G36Z

Cardiopulmonary Resuscitation	92950	5A12012, 5A19054, 5A1221Z
Source of procedures: Barnato AE, Farrell MH, Chang CC, Lave JR, Roberts MS, Angus DC. Development and validation of hospital "end-of-life" treatment intensity measures. Medical care. 2009;47(10):1098-105, linked to AACP coder website and Dartmouth Institute for Health Policy & Clinical Practice, 2016, “Our Parents, Ourselves: Healthcare for an Aging Population,” Dartmouth Atlas Project, Pg. 65, https://www.dartmouthatlas.org/downloads/reports/Our_Parents_Ourselves_021716.pdf<br>ICD-10 codes reported in Appendix E of Wasp GT, Alam SS, Brooks GA, Khayal IS, Kapadia NS, Carmichael DQ, et al. End-of-life quality metrics among Medicare decedents at minority-serving cancer centers: A retrospective study. Cancer Med. 2020;9(5):1911-21.		

**Codes indicative of Serious Illness and Conditions (Denominator)**	
**Condition**	**ICD-10 Code**
Advanced cancer<br><br>**Require 2 of these codes per NCI-SEER methodology.**	[ICD-10 liver excluding HCC (C22.1-4), esophagus (C15.3-5, C158-9), stomach (C16.0-6, C16.8-9), pancreas (C25.0-3, C25.7-9), peritoneum (C48.0-2, C48.8), brain (C71, C71.0-9), secondary malignant neoplasms (C78.00, C78.1-2, C78.39, C78.4-7, C78.89, C79.00, C79.11, C79.19, C79.2, C79.31-2, C79.49, C79.51-2, C79.60, C79.70, C79.81, C79.82, C79.89, C79.9), malignant pleural effusion (J91.0), malignant ascites (R18.0), disseminated malignant neoplasm (C80.0), leptomeningeal carcinomatosis (G96.12), heme malignancy (C81.09, C81.19, C81.29, C81.39, C81.49, C81.79, C81.99, C85.19, C85.29, C85.89, C91.02, C91.12, C91.52, C91.62, C91.92, C91.A2, C92.02, C92.12, C95.12)]
Advanced heart failure: Heart failure AND hospitalization<br><br>**Heart failure has been verified by requiring instances of at least 2 of these codes.**	ICD-10 (I09.81, I11.0, I13.0, I13.2, I50.1, I50.9, I50.20-23, I50.30-33, I50.40-43) in a code for an ambulatory visit AND a code for a hospitalization
Chronic obstructive lung disease AND oxygen OR hospitalization	ICD-10 (J43.9, J44.0, J44.1, J44.9) outpatient code AND ICD-10 (J95.850, Z99.1, Z99.11-12, Z99.81) OR ICD-10 (J43.9, J44.0, J44.1, J44.9) outpatient code AND hospitalization with COPD ICD code (J43.9, J44.0, J44.1, J44.9)
Cirrhosis AND Hepatic decompensation	ICD-10 (K70.3, K70.30, K70.31, K70.4, K70.40, K70.41, K74.3-5, K74.60, K74.69) AND [ICD-10 (K65.9, K67, K65.0, K65.2, K65.8); esophageal varices: ICD-9 (456, 456.2, 456.21); ICD-10 (I85.01, I85.10, I85.11); ascites: ICD-9 (789.5, 789.51, 789.59); ICD-10 (R18.8).]
End-stage renal disease<br><br>**Require 2 of these codes.**	ICD-10 (N18.5, N18.6, Z94.0, Z99.2, Z91.15, Z49.31, Z49.01, Z49.02, Z49.32) = ambulatory codes only
ALS<br><br>**Requires 2 instances of this code.**	ICD-10 (G12.21).
Dementia	ICD-10 (F01.50, F01.51, F02.80, F02.81, F03.90, F03.91, G30.0, G30.1, G30.9, G31.01, G31.09, G31.83, G31.84, R41.81)
Source: Walling AM, Sudore RL, Bell D, Tseng CH, Ritchie C, Hays RD, et al. Population-Based Pragmatic Trial of Advance Care Planning in Primary Care in the University of California Health System. Journal of palliative medicine. 2019;22(S1):72-81. (PMID: 31486723), modified to incorporate ICD-10 codes for dementia.	

9.4.2 Secondary outcomes (Note that this section refers to “Assessment of Implementation” as opposed to quantitative examination of secondary outcomes)

We characterize baseline primary care practice context and assess and triangulate stakeholder perspectives of SHARING Choices during and after the trial to identify barriers and facilitators that may affect sustainability and dissemination. The

approaches and measures that will be used to examine implementation are summarized in the table below.

With respect to *qualitative* data, skilled interviewers will conduct in-depth interviews with 20 patient-family dyads who engaged or did not engage in SHARING Choices (n=4 dyads at 5 practices) and all facilitators. We will purposefully sample 3-4 clinicians and 3-4 staff whose patients or work brings them into contact with the intervention protocol at 5 practices to participate in focus groups (n=5 focus groups; 1 per practice). Interview guides build on extensive previous qualitative, observational and mixed-methods work by our team and others in transforming primary care and improving ADRD patient-family communication and ACP. The stakeholder interviews will address not only "*does SHARING Choices work?*" but rather "*how and in what contexts does SHARING Choices work or can it be amended to work?*" In-depth interviews and focus groups will assess process measures of effectiveness by exploring the SHARING Choices intervention along the Consolidated Framework for Implementation Research (CFIR) domains.[86] These include stakeholder perception of the relative advantage, complexity, cost, quality, and compatibility with practice and health system goals. Concurrent content analysis will be used to iteratively review and adapt the interviews in a way to explore themes that emerge in early interviews with subsequent interviewees. [87-89] Sample size is based on achieving saturation of themes, or the point where little to no new information is being revealed. Saturation is generally achieved after 20-30 interviews per stakeholder group. Thus, we will aim to recruit at least 20 individuals from each stakeholder group at each health system to evaluate process and contextual factors impacting the effectiveness of the SHARING Choices intervention.

**Measures of Implementation by REAIM Domain**<br>(Note: measures encompass both Quantitative and *Qualitative* collection methods)
**Reach**<br>• Number and descriptive characteristics of practices included<br>• Number and descriptive characteristics of patients receiving each component of intervention (both all older adults, and those with an ADRD diagnosis). This information will be assessed using such measures as numbers of documented and billed ACP conversations and the percentage of patients who have registered for the patient portal themselves and via proxy/shared access.<br>• Number and descriptive characteristics of clinicians at SHARING Choices practices
**Effectiveness**<br>• *Perceived effectiveness of the SHARING Choices to improve ACP, advance directive, portal use, communication from the patient, clinician, and practice staff perspectives*
**Adoption**<br>• Descriptive characteristics of implementation teams and activities<br>• *Perceptions of SHARING Choices strategies implemented*<br>• *Practice readiness for SHARING Choices*<br>• *Alignment of SHARING Choices with organizational performance metrics and priorities*
**Implementation**<br>• *Implementation feasibility and practice and system stakeholder perceptions (receptivity)*
**Maintenance**<br>• *Interest in sustaining SHARING Choices beyond the grant period*<br>• *Perceived effectiveness of strategy on patient outcomes*

- *Modifications or adaptations necessary for sustained implementation*

9.5 Data Analyses

We compare SHARING Choices and control practices by analyzing the distribution of practice-level characteristics at baseline using appropriate graphical procedures, summary statistics, and multivariate methods. We will examine consistency of effects by patient age group, sex, race, ethnicity, ADRD diagnosis, comorbidity, primary care practice location (urban/rural/suburban), primary care state (MD, DC, VA), primary care size, and practice. We will compute the effect size of outcomes to assess SHARING Choices effects relative to published treatment effect estimates for ACP.

**ADRD Diagnosis Codes**	
**ICD-10 Code**	**ADRD Diagnosis**
F01.50, F01.51	Vascular dementia with or without behavioral disturbances
F02.80, F02.81	Dementia in other diseases classified elsewhere with or without behavioral disturbances
F03.90, F03.91	Unspecified dementia with or without behavioral disturbances
G30.0, G30.1, G30.9	Alzheimer's disease with early or late onset, or unspecified
G31.01, G31.09, G31.83, G31.84	Other degenerative diseases of nervous system, not elsewhere classified, Pick's disease, Other frontotemporal dementia, Dementia with Lewy bodies, Mild cognitive impairment
R41.81	Age-related cognitive decline
Source: Adapted from: Alzheimer's Association, 2018, Cognitive Assessment and Care Planning Services: Alzheimer's Association Expert Task Force Recommendations and Tools for Implementation (to drop G31.84; Mild Cognitive Impairment), https://www.alz.org/media/documents/cog-impair-assess-care-plan-code.pdf	

We test the effects of SHARING Choices on two groups: 1.) patients ages 65+ and 2.) patients with a diagnosis of ADRD, defined by ICD-10 diagnosis codes that are noted in the above table. A patient will be designated as having dementia based on the presence of any dementia diagnosis code any time before trial entry, and up to 1 year following this point. For both groups, we hypothesize that patients at intervention practices will be more likely to have a documented advance directive and/or MOLST in their electronic health record than patients at control practices at 12 months. We additionally hypothesize that among patients who die, those at intervention (versus control) practices will be less likely to experience potentially burdensome end-of-life care within 6 months of death. Potentially burdensome end-of-life care will be assessed in patients who die between 0 and 18 months after initial trial entry. For patients at intervention clinics, this focuses analysis on end-of-life care within 6 months of death that does not precede exposure to the intervention. All analyses will be performed using SAS statistical software and account for the cluster design, with the patient as the unit of analysis. All primary analyses comparing the effect of SHARING Choices to usual care will follow the principle of intent-to-treat, including all eligible study subjects with measurements at all available time points as appropriate to the outcome. The primary independent variable will be group assignment at the initial time point that eligible candidate patients are identified, consistent with intention to treat. To this end, a patient who changes care from intervention to control practice over the course of the study period would be assigned to the intervention practice, and those who are initially identified as eligible in control

practices and who later change care to an intervention practice would maintain their assignment to the control condition.

Comparability of the intervention and control groups will be assessed by comparing the distribution of practice-level and patient-level characteristics at baseline using appropriate graphical procedures, summary statistics, and multivariate methods. The study is designed to produce balance on important covariates at the practice level (unit of randomization) but not necessarily at the patient level. Baseline patient and practice characteristics that differ between groups will be included in our models of between-group differences. We will use SAS Proc GLIMMIX to examine primary outcomes that are binary (end-of-life preferences documentation at 12 months; receipt of potentially burdensome care within 6 months of death). Practice variables will be included in the model as fixed effect practice-level covariates. Initial analyses will examine distributions of baseline variables and changes by intervention and control group. We will build multilevel models that account for practice-level randomization and group assignment as well as baseline patient and practice covariates. In addition to overall effects on our primary outcome, we seek to examine consistency of effects that are stratified by patient age group (< 65, ≥ 65), ADRD diagnosis, and primary care practice. We will also evaluate the impact of the intervention on reducing disparities in care between whites and Blacks. We will compute the effect size of primary outcomes to assess intervention effects relative to published treatment effect estimates for ACP interventions.

As a supplemental analysis to the potentially burdensome end-of-life care analyses, we will examine whether rates of all-cause mortality differ across intervention and control groups. We are not hypothesizing that the SHARING Choices intervention will either prolong or shorten patients' lives significantly, but this will be examined empirically using Cox proportional hazards models. Patient-level covariates will include age at enrollment, gender, race, and ADRD diagnosis. These covariates will be entered into the model along with the intervention vs. control group contrast. We will use SAS Proc PHREG to conduct this analysis. The robust sandwich estimate option will be used to account for clustering. [91] Adjusted hazard ratios and 95% confidence intervals will be reported and interpreted.

For measures of implementation, we will use a convergent parallel mixed methods approach. For quantitative measures, we will assess implementation by evaluating uptake, mode and duration of intervention contacts, and variation by practice and patient characteristics, for all patients and those with ADRD. Among candidate patients from primary care practices randomized to SHARING Choices, we comparatively characterize those who did and did not engage in therapeutic components (uptake of the patient portal, participation in one or more advance care planning conversations) by age group, gender, race, ADRD diagnosis, primary care practice, and health system. We examine the acceptability of SHARING Choices and the mechanisms of observed effects.[92,93] Post-intervention focus groups and in-depth interviews will be audio-recorded, transcribed verbatim, and entered into MAXQDA textual data analysis software. The study team will conduct qualitative analyses concurrent to data collection using a framework approach[93,94] involving familiarization with content, identification of a thematic analytic

framework, data indexing, charting to abstract and distill key themes, and mapping of charts to interpret themes and context-specific meaning. We will ensure rigor and minimize bias by comprehensively documenting data collection, regular study team debriefs, triangulation of diverse stakeholder perspectives and data sources, attention to contradictory data elements, evaluating inter-rater agreement and feedback from health system stakeholders.[92,95] We will triangulate findings using standard methods to incorporate similarities and differences from varying perspectives.[96] We will cross-analyze findings from qualitative analyses with quantitative analyses of effectiveness for convergence to explore contextual impacts of settings and mechanisms through which intervention effects were observed and to identify how the intervention might be maintained, adapted to other settings and disseminated.

10 DATA COLLECTION AND QUALITY ASSURANCE

10.1 Data Collection Forms

Study outcomes will be quantitively examined using secondary data collected in routine clinical care. Research staff will extract outcome measures of ACP documentation, end-of-life preferences documentation, portal registration, and potentially burdensome end-of-life care from the patient electronic health record and the regional health information exchange (CRISP). Data extracted will also include demographics, diagnoses, comorbidity and location-based deprivation indices, and uptake of patient and family portal registration and use. All data are routinely recorded as part of routine clinical care and available in the electronic health record and/or through CRISP. Data will be de-identified for analyses and aggregated by practice.

No data collection forms will be used to document stakeholder interviews. Stakeholder interviews will be digitally recorded and transcribed for concurrent content analysis. Interview recordings and de-identified transcriptions will be maintained on a server accessible only to the study team. Thematic analysis will be completed, and themes will be presented in aggregate by stakeholder group.

10.2 Data Management

At the conclusion of the study, outcome measures will be extracted from the electronic health record and the Maryland-DC health information exchange (CRISP). The study team will separately provide (by participating health system) CRISP the medical record numbers for eligible patients who are under the care of clinicians at intervention and control primary care practices. CRISP will link individual-level health services utilization and strip medical record numbers and identifying individual-level information. CRISP will provide an anonymized file that includes health services use (see 9.4.1) for eligible patients by health system and intervention group (SHARING Choices or usual care). Information extracted from the Chesapeake Regional Information System (CRISP) and electronic health records will be stored in a database on a secure server. The data will be analyzed and presented in aggregate form at the practice level. No personal identifiers will be included in the database. All qualitative data, including audio recordings, transcripts and notes, will be similarly stored. Electronic analytic datasets will be provided to authorized study personnel with the same data protection requirements

established for the study database and can only be used on the SAFE Desktop platform. Only the PIs, Co-Investigators and research staff will have access to the SAFE Desktop site that contains study data. The PIs and the research staff are listed in eIRB and have completed the Human Subjects Training.

10.3 Quality Assurance

10.3.1 Training

ACP facilitators will be trained in all elements of SHARING Choices, including: The Respecting Choices First Steps curriculum, documenting advance directives, facilitating family meetings, communicating difficult news, and assessing cognitive capacity. Training will be delivered and reinforced using traditional didactics, case scenarios, and modeling and mentored role play (Supplemental Appendix II). Interventionists will be required to successfully facilitate at least one ACP conversation prior to facilitating ACP conversations and will receive regular mentoring, monitoring and feedback (See 5.3). The training schedule and plan for supervision and oversight of ACP facilitators will be tailored to each practice with input from the study team, implementation workgroup and practice administration.

We rely on an established curriculum, Respecting Choices http:// respecting-choices.org, to train ACP facilitators to lead advance care planning conversations. Respecting Choices includes: patient and family education materials, a structured educational curriculum to train non-physicians in the competencies of advance care planning and standardization of policies for embedding ACP in routine care delivery.[36,37] The Respecting Choices program includes 6 online modules with scripted interview tools and communication techniques to facilitate understanding ACP, exploring personal values, identifying a health care decision-maker, and communicating preferences for end-of-life care.

ACP facilitator quality and consistency with the Respecting Choices model of ACP will be completed in several ways. First, facilitators will participate in a weekly huddle with the expert facilitators and local Respecting Choices instructors. ACP facilitators will initially engage in weekly role-play scenarios and case reports to demonstrate maintenance of skills, the regularity of these meetings will fluctuate based on study phase and demonstrated facilitator competence. This will be completed via Teams and/or Zoom and facilitated by local Respecting Choices instructors and study investigators. Finally, Facilitators will be required to participate in a scored interaction with simulated patients to assess fidelity to ACP training. Additional booster training will be offered for those who score below pre-determined thresholds.

Study staff training includes instruction on the intervention and supplementary background literature, ADRD, data safety protocols, quality control procedures, and study procedures and reporting. Upon satisfaction of training requirements and review by study leadership, staff must provide documented completion of HIPAA and human subject training.

Program Orientation will be designed as a practice in-service at the practice. Since the program is being deployed as a system-level initiative the presentations will address the problem the program is attempting to help with -- the system-level needs assessment related to low documentation of end-of-life preferences in the electronic health record -- the program components and proposed workflows, planning support and timeline, and resources and support for implementation and program monitoring. An overarching presentation and planning toolkit drafted from R61 activities will be deployed at each system. Upon receipt of our notice of award for the R33 trial and the identification of intervention practices, the study team will work with practice leaders to tailor materials to address practice-level workflows, staffing, and practice needs. This approach will enhance adoption and implementation decision making by clear demonstration of the *relative advantage* for each practice.

Practice Facilitation: Practice staff will be oriented to the SHARING Choices intervention including the process of identifying and contacting patients eligible for the program and documents or messages to be sent to patients (portal access, advance directive, SHARING Choices checklist, letter), process for uploading documents into the electronic health record or facilitating access (*adapted to meet each practice's workflow*), and orientation to advance care planning and advance directives and their importance and ADRD.

Electronic Health Record Documentation. ACP facilitators will be trained on how to screen the chart for evidence of ADRD, conduct a pre-ACP chart review, document the ACP conversation in the electronic health record, and notify the clinician that the patient/family member has a completed ACP conversation, including current status of advance directive documentation. Charting standards will be established and tailored to the needs of each practice and clinicians and staff will be oriented to the process.

Clinician Orientation and Workflow: In the R61 phase we learned that clinician orientation needs to be highly tailored to the practice context. Clinician in-services at each practice will be led by a local implementation team who will orient clinicians to the proposed workflow, clinician-level data for documentation of end-of-life preferences, an orientation to the Respecting Choices™ curriculum and scope of practice for the ACP facilitators. Clinicians will be oriented to the SHARING Choices agenda setting checklist and letter. Adaptations to the letter and workflow will be documented and added to a fidelity checklist for each practice.

10.3.2 Quality Control Committee

The study will not assemble a Quality Control Committee. Study processes will be developed to ensure the quality of data collection and fidelity to assessment schedules (see 5.3, 10.3.4, 10.3.5 and Appendix 5D).

10.3.3 Metrics

As a pragmatic trial, quality control will be monitored through data collected in routine care and ongoing interactions with ACP facilitators and practice staff (See 5.3).

10.3.4 Protocol Deviations

Not applicable.

10.3.5 Monitoring

ACP facilitators from both health systems will be regularly convened to review conversations, troubleshoot issues and answer questions. After each in-person advance care planning meeting, facilitators will document their impressions of the meeting content and progress in the electronic health record. This documentation will include details about the structure of the meeting (individuals present, location, meeting duration, etc.).

The study team will convene facilitators weekly during the first six months of the study, and then monthly thereafter. Holding supervision sessions with facilitators will afford the opportunity to discuss concerns noted in ACP conversation field notes and review of field observations. We will seek to maintain delivery skill and identify potential drift, and remediation and retesting in response to identified deficiencies or drift in quality of intervention delivery.

As noted in 5.2. the study team will work with each of the 19 intervention practices to develop processes to monitor study processes, including identifying targets and metrics of success. Chair of the ACP Facilitator Training and Oversight Workgroup will be in regular contact with facilitators and will track feedback regarding practice-level systems and workflows that are working well or that pose barriers to maintaining fidelity to study processes. ACP facilitators will additionally be asked to regularly document field observations which will be shared with the study team.

11 PARTICIPANT RIGHTS AND CONFIDENTIALITY

11.1 Institutional Review Board (IRB) Review

This protocol and any subsequent modifications will be reviewed and approved by the IRB or ethics committee responsible for oversight of the study.

11.2 Informed Consent Forms

There will be no consent forms for this study as this is a practice-level pragmatic trial and all components will be available to all patients over 65 years of age at intervention primary care practices. In Year 5 (2022) we will conduct structured qualitative interviews with clinicians, patient-family dyads, and ACP facilitators to determine factors related to the implementation of SHARING Choices. We expect that participants will complete oral consent forms for this aspect of the study.

11.3 Participant Confidentiality

Any data that is electronically extracted will be manipulated solely in an encrypted, access-restricted virtual environment to maintain confidentiality. All patient information will be stored on an encrypted virtual workstation (SAFE Desktop) on servers maintained at Johns Hopkins. SAFE Desktop will be used for all patient identification

and data analyses. Information will not be released, except as necessary for monitoring by IRB, the FDA, the NIA, and the OHRP.

We will institute multiple procedures for protecting against and minimizing risks to privacy and confidentiality. Staff will be trained regarding HIPAA and human subjects protections regulations and procedures. No data will be stored or analyzed on portable devices such as laptops, flash drives, smart phones, or personal digital assistants.

11.4 Study Discontinuation

The study may be discontinued at any time by the IRB, the NIA, the OHRP or other government agencies as part of their duties to ensure that research participants are protected.

12 ETHICAL CONSIDERATIONS

This study is guided by the prevailing ethical considerations that are outlined in the 1974 Belmont report that include: respect for persons, justice, and beneficence. These principles are outlined in Good Clinical Practice training criteria that have been completed by the study team and are supported by this protocol and our study team's standard operating procedures which ensure fidelity to the protocol.

This is a minimal risk study. Given the evidence supporting the components of SHARING Choices, we anticipate that older persons, including those with ADRD, and family will experience more benefit than risk from being in a practice that has SHARING Choices and that risks associated with participation are reasonable in comparison to knowledge that may be gained. Benefits for patients in SHARING Choices practices may include greater clarity regarding the patient's health and treatment preferences and the communication roles to be assumed by family during face-to-face medical visits, in electronic interactions with primary care providers, and in future medical decision-making. Societal benefits will result from this study. We will evaluate SHARING Choices to improve the quality of communication about end of life care in primary care, extending knowledge of regarding the implementation and effects of advance care planning for persons, including those with ADRD, outside of institutional settings. If this trial has a positive effect on communication, the methodology has broad potential application to improve advance care planning and end of life care in primary care. Participants in control practices will not directly benefit from participation.

13 PUBLICATION OF RESEARCH FINDINGS

This study will be conducted in accordance with the following publication and data sharing policies and regulations. National Institutes of Health (NIH) Public Access Policy, which ensures that the public has access to the published results of NIH funded research. It requires scientists to submit final peer-reviewed journal manuscripts that arise from NIH funds to the digital archive PubMed Central upon acceptance for publication. This study will comply with the NIH Data Sharing Policy and Policy on the Dissemination of NIH-Funded Clinical Trial Information and the Clinical Trials Registration and Results Information Submission rule. As such, this trial will be registered at ClinicalTrials.gov, and results information from this trial will be submitted to ClinicalTrials.gov. In addition, every attempt will be made to publish results in peer-reviewed journals.

14 REFERENCES

1. Hurd MD, Martorell P, Delavande A, Mullen KJ, Langa KM. Monetary costs of dementia in the United States. *The New England journal of medicine.* 2013;368(14):1326-1334.
2. CDC. Health, United States, 2017. U.S. Government Printing Office. https://www.cdc.gov/nchs/data/hus/hus17.pdf. Published 2017. Accessed 12/23/2018.
3. Silveira MJ, Kim SY, Langa KM. Advance directives and outcomes of surrogate decision making before death. *The New England journal of medicine.* 2010;362(13):1211-1218.
4. Sessums LL, Zembrzuska H, Jackson JL. Does This Patient Have Medical Decision-Making Capacity? *Jama-J Am Med Assoc.* 2011;306(4):420-427.
5. Wendler D, Rid A. Systematic review: the effect on surrogates of making treatment decisions for others. *Annals of internal medicine.* 2011;154(5):336-346.
6. Cagle JG, McClymont KM, Thai JN, Smith AK. "If You Don't Know, All of a Sudden, They're Gone": Caregiver Perspectives About Prognostic Communication for Disabled Elderly Adults. *Journal of the American Geriatrics Society.* 2016;64(6):1299-1306.
7. Mitchell SL, Teno JM, Kiely DK, et al. The clinical course of advanced dementia. *The New England journal of medicine.* 2009;361(16):1529-1538.
8. Shalowitz D, Garrett-Mayer E, Wendler D. The accuracy of surrogate decision makers: a systematic review. *Archives of internal medicine.* 2006;166(5):493-497.
9. White DB, Ernecoff N, Buddadhumaruk P, et al. Prevalence of and Factors Related to Discordance About Prognosis Between Physicians and Surrogate Decision Makers of Critically Ill Patients. *JAMA : the journal of the American Medical Association.* 2016;315(19):2086-2094.
10. Harrison KL, Adrion ER, Ritchie CS, Sudore RL, Smith AK. Low Completion and Disparities in Advance Care Planning Activities Among Older Medicare Beneficiaries. *JAMA internal medicine.* 2016.
11. Wright AA, Zhang B, Ray A, et al. Associations between end-of-life discussions, patient mental health, medical care near death, and caregiver bereavement adjustment. *JAMA : the journal of the American Medical Association.* 2008;300(14):1665-1673.
12. Lakin JR, Block SD, Billings JA, et al. Improving Communication About Serious Illness in Primary Care: A Review. *JAMA internal medicine.* 2016.
13. Karlawish J, Casarett D, James B, Xie S, Kim S. The ability of persons with Alzheimer disease (AD) to make a decision about taking an AD treatment. *Neurology.* 2005;64(9):1514-1519.
14. Hirschman K, Xie S, Feudtner C, Karlawish J. How does an Alzheimer's disease patient's role in medical decision making change over time? *Journal of geriatric psychiatry and neurology.* 2004;17(2):55-60.
15. Hanson LC, Carey TS, Caprio AJ, et al. Improving decision-making for feeding options in advanced dementia: a randomized, controlled trial. *Journal of the American Geriatrics Society.* 2011;59(11):2009-2016.
16. Vick J, Amjad H, Smith KC, et al. "Let him speak:" A descriptive qualitative study of the roles and behaviors of family companions in primary care visits among older adults with cognitive impairment. *International journal of geriatric psychiatry.* 2018;33(1):e103-e112.
17. Volandes AE, Paasche-Orlow MK, Barry MJ, et al. Video decision support tool for advance care planning in dementia: randomised controlled trial. *Bmj.* 2009;338:b2159.

18. Hanson LC, Zimmerman S, Song MK, et al. Effect of the Goals of Care Intervention for Advanced Dementia: A Randomized Clinical Trial. *JAMA internal medicine.* 2016.
19. Hirschman KB, Kapo JM, Karlawish JH. Identifying the factors that facilitate or hinder advance planning by persons with dementia. *Alzheimer disease and associated disorders.* 2008;22(3):293-298.
20. Kasper JD, Freedman VA, Spillman BC, Wolff JL. The disproportionate impact of dementia on family and unpaid caregiving to older adults. *Health affairs.* 2015;34(10):1642-1649.
21. Friedman EM, Shih RA, Langa KM, Hurd MD. US Prevalence And Predictors Of Informal Caregiving For Dementia. *Health affairs.* 2015;34(10):1637-1641.
22. Association As. 2013 Alzheimer's disease: facts and figures. In:2013: http://www.alz.org/downloads/facts_figures_2013.pdf.
23. Wolff JL, Roter DL. Hidden in plain sight: Medical visit companions as a quality of care resource for vulnerable older adults. *Archives of internal medicine.* 2008;168(13):1409-1415.
24. Wolff JL, Roter DL. Family presence in routine medical visits: A meta-analytical review. *Social science & medicine.* 2011;72(6):823-831.
25. Wolff JL, Roter DL. Older adults' mental health function and patient-centered care: does the presence of a family companion help or hinder communication? *Journal of general internal medicine.* 2012;27(6):661-668.
26. van der Steen JT, van Soest-Poortvliet MC, Hallie-Heierman M, et al. Factors associated with initiation of advance care planning in dementia: a systematic review. *Journal of Alzheimer's disease : JAD.* 2014;40(3):743-757.
27. Sharp T, Moran E, Kuhn I, Barclay S. Do the elderly have a voice? Advance care planning discussions with frail and older individuals: a systematic literature review and narrative synthesis. *The British journal of general practice : the journal of the Royal College of General Practitioners.* 2013;63(615):e657-668.
28. Oczkowski SJ, Chung HO, Hanvey L, Mbuagbaw L, You JJ. Communication Tools for End-of-Life Decision-Making in Ambulatory Care Settings: A Systematic Review and Meta-Analysis. *PloS one.* 2016;11(4):e0150671.
29. Fetherstonhaugh D, McAuliffe L, Bauer M, Shanley C. Decision-making on behalf of people living with dementia: how do surrogate decision-makers decide? *Journal of medical ethics.* 2017;43(1):35-40.
30. Mitchell SL, Black BS, Ersek M, et al. Advanced dementia: state of the art and priorities for the next decade. *Annals of internal medicine.* 2012;156(1 Pt 1):45-51.
31. Jones K, Birchley G, Huxtable R, Clare L, Walter T, Dixon J. End of life care: A scoping review of experiences of Advance Care Planning for people with dementia. *Dementia.* 2016.
32. Hancock K, Clayton JM, Parker SM, et al. Truth-telling in discussing prognosis in advanced life-limiting illnesses: a systematic review. *Palliative medicine.* 2007;21(6):507-517.
33. McCabe M, You E, Tatangelo G. Hearing Their Voice: A Systematic Review of Dementia Family Caregivers' Needs. *The Gerontologist.* 2016;56(5):e70-88.
34. Peterson K, Hahn H, Lee AJ, Madison CA, Atri A. In the Information Age, do dementia caregivers get the information they need? Semi-structured interviews to determine

informal caregivers' education needs, barriers, and preferences. *BMC geriatrics.* 2016;16(1):164.
35. Luu NP, Nigrin C, Peairs K, et al. Increasing advance care planning completion at an academic internal medicine outpatient clinic. *Journal of pain and symptom management.* 2017;54(3):383-386.
36. Choices R. Return on Investment: Implementation of Respecting Choices Model of Advance Care Planning. In: System GH, ed. LaCrosse, WI: Gundersen Health.
37. Hammes BJ, Rooney BL. Death and end-of-life planning in one midwestern community. *Archives of internal medicine.* 1998;158(4):383-390.
38. Wolff JL, Roter DL, Barron J, et al. A tool to strengthen the older patient-companion partnership in primary care: Results from a pilot study. *Journal of the American Geriatrics Society.* 2014;62(2):312-319.
39. Wolff JL, Darer JD, Berger A, et al. Inviting patients and care partners to read doctors' notes: OpenNotes and shared access to electronic medical records. *Journal of the American Medical Informatics Association : JAMIA.* 2016.
40. Wolff JL, Berger A, Clarke D, et al. Patients, care partners, and shared access to the patient portal: online practices at an integrated health system. *Journal of the American Medical Informatics Association : JAMIA.* 2016;23(6):1150-1158.
41. NCI. Education in palliative and end-of-life care for oncology (EPEC-O) curriculum on cultural considerations when caring for African Americans. 2013.
42. Rocque GB, Dionne-Odom JN, Sylvia Huang CH, et al. Implementation and Impact of Patient Lay Navigator-led Advance Care Planning Conversations. *Journal of pain and symptom management.* 2017.
43. Detering KM, Hancock AD, Reade MC, Silvester W. The impact of advance care planning on end of life care in elderly patients: randomised controlled trial. *Bmj.* 2010;340:c1345.
44. Pecanac KE, Repenshek MF, Tennenbaum D, Hammes BJ. Respecting Choices(R) and advance directives in a diverse community. *Journal of palliative medicine.* 2014;17(3):282-287.
45. Huang CH, Crowther M, Allen RS, et al. A Pilot Feasibility Intervention to Increase Advance Care Planning among African Americans in the Deep South. *Journal of palliative medicine.* 2016;19(2):164-173.
46. Boettcher I, Turner R, Briggs L. Telephonic advance care planning facilitated by health plan case managers. *Palliative & supportive care.* 2015;13(3):795-800.
47. Rietjens JA, Korfage IJ, Dunleavy L, et al. Advance care planning--a multi-centre cluster randomised clinical trial: the research protocol of the ACTION study. *BMC cancer.* 2016;16:264.
48. Korfage IJ, Rietjens JA, Overbeek A, et al. A cluster randomized controlled trial on the effects and costs of advance care planning in elderly care: study protocol. *BMC geriatrics.* 2015;15:87.
49. Heiman H, Bates DW, Fairchild D, Shaykevich S, Lehmann LS. Improving completion of advance directives in the primary care setting: a randomized controlled trial. *The American journal of medicine.* 2004;117(5):318-324.
50. Mack JW, Cronin A, Keating NL, et al. Associations between end-of-life discussion characteristics and care received near death: a prospective cohort study. *Journal of*

clinical oncology : official journal of the American Society of Clinical Oncology. 2012;30(35):4387-4395.

51. Nicholas LH, Langa KM, Iwashyna TJ, Weir DR. Regional variation in the association between advance directives and end-of-life Medicare expenditures. *JAMA : the journal of the American Medical Association.* 2011;306(13):1447-1453.
52. Haywood K, Marshall S, Fitzpatrick R. Patient participation in the consultation process: a structured review of intervention strategies. *Patient education and counseling.* 2006;63(1-2):12-23.
53. Kinnersley P, Edwards A, Hood K, et al. Interventions before consultations to help patients address their information needs by encouraging question asking: systematic review. *Bmj.* 2008;337:a485.
54. Otte-Trojel T, de Bont A, Rundall TG, van de Klundert J. How outcomes are achieved through patient portals: a realist review. *Journal of the American Medical Informatics Association : JAMIA.* 2014;21(4):751-757.
55. Piette JD, Marinec N, Janda K, et al. Structured Caregiver Feedback Enhances Engagement and Impact of Mobile Health Support: A Randomized Trial in a Lower-Middle-Income Country. *Telemedicine journal and e-health : the official journal of the American Telemedicine Association.* 2015.
56. Piette JD, Striplin D, Marinec N, et al. A Mobile Health Intervention Supporting Heart Failure Patients and Their Informal Caregivers: A Randomized Comparative Effectiveness Trial. *Journal of medical Internet research.* 2015;17(6):e142.
57. Aikens JE, Trivedi R, Heapy A, Pfeiffer PN, Piette JD. Potential Impact of Incorporating a Patient-Selected Support Person into mHealth for Depression. *Journal of general internal medicine.* 2015;30(6):797-803.
58. Wittenberg-Lyles E, Oliver DP, Kruse RL, Demiris G, Gage LA, Wagner K. Family caregiver participation in hospice interdisciplinary team meetings: how does it affect the nature and content of communication? *Health communication.* 2013;28(2):110-118.
59. Piette JD, Gregor MA, Share D, et al. Improving heart failure self-management support by actively engaging out-of-home caregivers: results of a feasibility study. *Congest Heart Fail.* 2008;14(1):12-18.
60. Handley M, Bunn F, Goodman C. Dementia-friendly interventions to improve the care of people living with dementia admitted to hospitals: a realist review. *BMJ open.* 2017;7(7):e015257.
61. Graham J. New Toolkits Help Physicians Detect, Diagnose, and Manage Dementia. *JAMA : the journal of the American Medical Association.* 2017;318(14):1310-1312.
62. Administration DHRS. Training curriculum: Alzheimer's Disease and Related Dementias. https://bhw.hrsa.gov/grants/geriatrics/alzheimers-curriculum. Published 2017. Accessed.
63. Borson S, Chodosh J, Cordell C, et al. Innovation in care for individuals with cognitive impairment: Can reimbursement policy spread best practices? *Alzheimer's & dementia : the journal of the Alzheimer's Association.* 2017;13(10):1168-1173.
64. Reuben D, Levin J, Frank J, et al. Closing the dementia care gap: Can referral to Alzheimer's Association chapters help? *Alzheimer's & dementia : the journal of the Alzheimer's Association.* 2009;5(6):498-502.

65. Bellg AJ, Borrelli B, Resnick B, et al. Enhancing treatment fidelity in health behavior change studies: best practices and recommendations from the NIH Behavior Change Consortium. *Health Psychol.* 2004;23(5):443-451.
66. Nicholas LH, Bynum JP, Iwashyna TJ, Weir DR, Langa KM. Advance directives and nursing home stays associated with less aggressive end-of-life care for patients with severe dementia. *Health affairs.* 2014;33(4):667-674.
67. Mitchell SL, Teno JM, Roy J, Kabumoto G, Mor V. Clinical and organizational factors associated with feeding tube use among nursing home residents with advanced cognitive impairment. *JAMA : the journal of the American Medical Association.* 2003;290(1):73-80.
68. Gozalo P, Teno JM, Mitchell SL, et al. End-of-life transitions among nursing home residents with cognitive issues. *The New England journal of medicine.* 2011;365(13):1212-1221.
69. Walczak A, Butow PN, Bu S, Clayton JM. A systematic review of evidence for end-of-life communication interventions: Who do they target, how are they structured and do they work? *Patient education and counseling.* 2016;99(1):3-16.
70. Raudenbush SW, Liu X. Statistical power and optimal design for multisite randomized trials. *Psychological methods.* 2000;5(2):199-213.
71. Spybrook J, Raudenbush SW, Liu X, Congdon R, Martinez A. Optimal design for longitudinal and multilevel research: documentation for the "Optimal Design" software. *Ann Arbor: University of Michigan School of Education, Hierarchical Models Project.* 2006.
72. Hussain NA, McGuire M, Colburn JL. Advance care planning workflow implementation for older adults in primary care. Paper presented at: Society for General Internal Medicine2018; Denver CO.
73. Carey EC, Walter LC, Lindquist K, Covinsky KE. Development and validation of a functional morbidity index to predict mortality in community-dwelling elders. *Journal of general internal medicine.* 2004;19(10):1027-1033.
74. Huang J, Roth DL. Using the half normal distribution to quantify covariate balance in cluster-randomized pragmatic trials. *Trials.* 2021;in press.
75. Ciolino JD, Diebold A, Jensen JK, Rouleau GW, Koloms KK, Tandon D. Choosing an imbalance metric for covariate-constrained randomization in multiple-arm cluster-randomized trials. *Trials.* 2019;20(1):293.
76. Sudore R, Le GM, McMahan R, Feuz M, Katen M, Barnes DE. The advance care planning PREPARE study among older Veterans with serious and chronic illness: study protocol for a randomized controlled trial. *Trials.* 2015;16:570.
77. Bernacki R, Hutchings M, Vick J, et al. Development of the Serious Illness Care Program: a randomised controlled trial of a palliative care communication intervention. *BMJ open.* 2015;5(10):e009032.
78. Tarzian AJ, Cheevers NB. Maryland's Medical Orders for Life-Sustaining Treatment Form Use: Reports of a Statewide Survey. *Journal of palliative medicine.* 2017.
79. NPPTF. National POLST Paradigm: Appropriate POLST Paradigm Form Use Policy. In. *polst.org.* Vol 2017: National Polst Paradigm Task Force; 2017.
80. Sudore RL, Fried TR. Redefining the "planning" in advance care planning: preparing for end-of-life decision making. *Annals of internal medicine.* 2010;153(4):256-261.

81. Bernacki RE, Block SD, for the American College of Physicians High Value Care Task F. Communication About Serious Illness Care Goals: A Review and Synthesis of Best Practices. *JAMA internal medicine.* 2014.
82. IOM. *Dying in America: Improving Quality and Honoring Individual Preferences Near the End of Life.* Washington DC: National Academy Press; 2014.
83. Walling AM, Sudore RL, Bell D, et al. Population-Based Pragmatic Trial of Advance Care Planning in Primary Care in the University of California Health System. *Journal of palliative medicine.* 2019;22(S1):72-81.
84. Bomba PA, Kemp M, Black JS. POLST: An improvement over traditional advance directives. *Cleveland Clinic journal of medicine.* 2012;79(7):457-464.
85. Barnato AE, Farrell MH, Chang CC, Lave JR, Roberts MS, Angus DC. Development and validation of hospital "end-of-life" treatment intensity measures. *Medical care.* 2009;47(10):1098-1105.
86. Damschroder L, Aron D, Keith S, Kirsh J, Alexander B, Lowery J. Fostering implementation of health services research findings into practice: a consolidated framework for advancing implementation science. *Implementation science : IS.* 2009;50(4).
87. Moore GF, Audrey S, Barker M, et al. Process evaluation of complex interventions: Medical Research Council guidance. *Bmj.* 2015;350:h1258.
88. Cresswell JW, Plano Clark VL. *Designing and conducting mixed methods research.* Thousand Oaks, CA: SAGE Publications, Inc.; 2018.
89. Coffey A. *Making sense of qualitative data: Complementary research strategies.* Thousand Oaks, CA: SAGE; 1996.
90. Round J. *Care at the End of Life: An Economic Perspective.* Switzerland: Springer; 2016.
91. Gharibvand L, Liu L. Analysis of survival data with clustered events. 2009.
92. Palinkas LA. Qualitative and mixed methods in mental health services and implementation research. *Journal of clinical child and adolescent psychology : the official journal for the Society of Clinical Child and Adolescent Psychology, American Psychological Association, Division 53.* 2014;43(6):851-861.
93. Pope C, Ziebland S, Mays N. Qualitative research in health care. Analysing qualitative data. *Bmj.* 2000;320(7227):114-116.
94. Gale NK, Heath G, Cameron E, Rashid S, Redwood S. Using the framework method for the analysis of qualitative data in multi-disciplinary health research. *BMC medical research methodology.* 2013;13:117.
95. Mays N, Pope C. Qualitative research in health care. Assessing quality in qualitative research. *Bmj.* 2000;320(7226):50-52.
96. Farmer T, Robinson K, Elliott SJ, Eyles J. Developing and implementing a triangulation protocol for qualitative health research. *Qualitative health research.* 2006;16(3):377-394.
