# Supplementary material for: Advance Care Planning, End-of-Life Preferences, and Burdensome Care: A Pragmatic Cluster Randomized Clinical Trial
Source: JAMA Intern Med. 2024 Dec 2;185(2):162–70. doi: 10.1001/jamainternmed.2024.6215 (PMC11612918; doi:10.1001/jamainternmed.2024.6215)
Supplement: Supplement 2. — eTable 1. Construction of Analytic Cohort for Analysis of Potentially Burdensome Care at End of Life eTable 2. Potentially Burdensome End-of-Life Care in Patients With a Serious Illness 6 Months or Before Death by Treatment Group, Procedure, and Subgroup eTable 3. Documentation of New and Updated End-of-Life Preferences by Type and Subgroup eTable 4. Potentially Burdensome End-of-Life Care Among Older Decedents With Serious Illness, 6 to 18 Months After Study Entry eFigure 1. Proportion of Older Adults Who Engaged in ACP at 19 Intervention Primary Care Practices eFigure 2. New EHR-Documented End-of-Life Preferences by ACP at 19 Primary Care Practices [file jamainternmed-e246215-s002.pdf]

Supplemental Online Content

Wolff JL, Scerpella D, Giovanetti ER, et al; SHARING Choices Investigators. Advance care planning, end-of-life preferences, and burdensome care: a pragmatic cluster randomized clinical trial. *JAMA Intern Med*. Published online December 2, 2024.
doi:10.1001/jamainternmed.2024.6215

eTable 1. Construction of Analytic Cohort for Analysis of Potentially Burdensome Care at End of Life

eTable 2. Potentially Burdensome End-of-Life Care in Patients With a Serious Illness 6 Months or Before Death by Treatment Group, Procedure, and Subgroup

eTable 3. Documentation of New and Updated End-of-Life Preferences by Type and Subgroup

eTable 4. Potentially Burdensome End-of-Life Care Among Older Decedents With Serious Illness, 6 to 18 Months After Study Entry

eFigure 1. Proportion of Older Adults Who Engaged in ACP at 19 Intervention Primary Care Practices

eFigure 2. New EHR-Documented End-of-Life Preferences by ACP at 19 Primary Care Practices

This supplementary material has been provided by the authors to give readers additional information about their work.

© 2024 Wolff JL et al. *JAMA Internal Medicine*.

eTable 1. Construction of Analytic Cohort for Analysis of Potentially Burdensome Care at End of Life

Panel A

**Observation Period**	**Deaths; n (column %)**
0 to 6 Months After Study Entry	717 (25.9%)
6 to 12 Months After Study Entry	847 (30.6%)
12 to 18 Months After Study Entry	707 (25.5%)
18 to 24 Months After Study Entry	475 (17.1%)
24+ Months After Study Entry	26 (0.9%)
Total Deaths Reported by CRISP	2,772 (100.0%)

Note: The 2,271 deaths corresponding with 0-18 months following study entry comprises the cohort of focus for our outcome of potentially burdensome care within 6 months of death. A total of 48 deaths occurred among non-Maryland residents; 43 of these occurred during the observation window of 0-18 months after study entry.

Panel B

**Decedents with Serious Illness at least 6 Months Prior to Death**	**SHARING Choices n (%)**	**Control n (%)**
Cancer	71 (9.1%)	123 (8.3%)
Heart failure	302 (38.7%)	479 (32.2%)
Chronic obstructive lung disease	203 (26.0%)	330 (22.2%)
Cirrhosis and Hepatic Decompensation	11 (1.4%)	24 (1.6%)
End-Stage Renal Disease	60 (7.7%)	116 (7.8%)
Amyotrophic Lateral Sclerosis	1 (0.1%)	2 (0.1%)
Alzheimer's Disease and Related Dementias (ADRD)	181 (23.2%)	409 (27.5%)
**Any Serious Illness****	521 (66.7%)	977 (65.6%)

Note: The subset of 1,498 decedents involving Maryland residents with serious illness diagnoses 6 months or more preceding death (as operationalized in Panel C) who died within 18-months of study entry as described in Panel A (1,498 of 2,271, 66.0% of the total sample; 521 of 781, 66.7%, in the SHARING Choices group and 977 of 1,490, 65.6%, in the Control Group) comprise the analytic sample for our outcome of potentially burdensome care within 6 months of death.

Panel C

Codes Used to Identify Serious Illness and Conditions

Advanced cancer<br><br>**Require 2 of these codes per NCI-SEER methodology.**	[ICD-10 liver excluding HCC (C22.1-4), esophagus (C15.3-5, C158-9), stomach (C16.0-6, C16.8-9), pancreas (C25.0-3, C25.7-9), peritoneum (C48.0-2, C48.8), brain (C71, C71.0-9), secondary malignant neoplasms (C78.00, C78.1-2, C78.39, C78.4-7, C78.89, C79.00, C79.11, C79.19, C79.2, C79.31-2, C79.49, C79.51-2, C79.60, C79.70, C79.81, C79.82, C79.89, C79.9), malignant pleural effusion (J91.0), malignant ascites (R18.0), disseminated malignant neoplasm (C80.0), leptomeningeal carcinomatosis (G96.12), heme malignancy (C81.09, C81.19, C81.29, C81.39, C81.49, C81.79, C81.99, C85.19, C85.29, C85.89, C91.02, C91.12, C91.52, C91.62, C91.92, C91.A2, C92.02, C92.12, C95.12)]

© 2024 Wolff JL et al. *JAMA Internal Medicine*.

Advanced heart failure: Heart failure AND hospitalization<br><br>**Heart failure has been verified by requiring instances of at least <u>2 of these codes</u>.**	ICD-10 (I09.81, I11.0, I13.0, I13.2, I50.1, I50.9, I50.20-23, I50.30-33, I50.40-43) in a code for an ambulatory visit AND a code for a hospitalization
Chronic obstructive lung disease AND oxygen OR hospitalization	ICD-10 (J43.9, J44.0, J44.1, J44.9) outpatient code AND ICD-10 (J95.850, Z99.1, Z99.11-12, Z99.81) OR ICD-10 (J43.9, J44.0, J44.1, J44.9) outpatient code AND hospitalization with COPD ICD code (J43.9, J44.0, J44.1, J44.9)
Cirrhosis AND Hepatic decompensation	ICD-10 (K70.3, K70.30, K70.31, K70.4, K70.40, K70.41, K74.3-5, K74.60, K74.69) AND [ICD-10 (K65.9, K67, K65.0, K65.2, K65.8); esophageal varices: ICD-9 (456, 456.2, 456.21); ICD-10 (I85.01, I85.10, I85.11); ascites: ICD-9 (789.5, 789.51, 789.59); ICD-10 (R18.8).]
End-stage renal disease<br><br>**Require <u>2 of these codes</u>.**	ICD-10 (N18.5, N18.6, Z94.0, Z99.2, Z91.15, Z49.31, Z49.01, Z49.02, Z49.32) = ambulatory codes only
ALS<br><br>**Requires <u>2 instances of this code</u>.**	ICD-10 (G12.21).
Dementia	ICD-10 (F01.50, F01.51, F02.80, F02.81, F03.90, F03.91, G30.0, G30.1, G30.9, G31.01, G31.09, G31.83, G31.84, R41.81)
Advanced cancer<br><br>**Require 2 of these codes per NCI-SEER methodology.**	[ICD-10 liver excluding HCC (C22.1-4), esophagus (C15.3-5, C158-9), stomach (C16.0-6, C16.8-9), pancreas (C25.0-3, C25.7-9), peritoneum (C48.0-2, C48.8), brain (C71, C71.0-9), secondary malignant neoplasms (C78.00, C78.1-2, C78.39, C78.4-7, C78.89, C79.00, C79.11, C79.19, C79.2, C79.31-2, C79.49, C79.51-2, C79.60, C79.70, C79.81, C79.82, C79.89, C79.9), malignant pleural effusion (J91.0), malignant ascites (R18.0), disseminated malignant neoplasm (C80.0), leptomeningeal carcinomatosis (G96.12), heme malignancy (C81.09, C81.19, C81.29, C81.39, C81.49, C81.79, C81.99, C85.19, C85.29, C85.89, C91.02, C91.12, C91.52, C91.62, C91.92, C91.A2, C92.02, C92.12, C95.12)]
Based on ICD codes identified by AM Walling et al, (2019). "Population-Based Pragmatic Trial of Advance Care Planning in Primary Care in the University of California Health System." <u>J Palliat Med</u> **22**(S1): 72-81; adapted to include ADRD following: Alzheimer's Association. (2017) "Medicare's cognitive impairment assessment and care planning codes".	

© 2024 Wolff JL et al. *JAMA Internal Medicine*.

eTable 2. Potentially Burdensome End-of-Life Care in Patients With a Serious Illness 6 Months or Before Death by Treatment Group, Procedure, and Subgroup

**Procedure**	**SHARING Choices n (%)**	**Control n (%)**
**Full Cohort**	521	977
Intubation/Mechanical Ventilation	99 (19.0%)	122 (12.5%)
Tracheostomy	5 (1.0%)	8 (0.8%)
GI Tube Insertion	12 (2.3%)	23 (2.4%)
Hemodialysis	48 (9.2%)	73 (7.5%)
Enteral/Parenteral Nutrition	13 (2.5%)	15 (1.5%)
CPR	36 (6.9%)	51 (5.2%)
**Any (1+) Procedure**	150 (28.8%)	204 (20.9%)
**Decedents with Dementia Diagnosis**	181	409
Intubation/Mechanical Ventilation	21 (11.6%)	33 (8.1%)
Tracheostomy	0 (0.0%)	2 (0.5%)
GI Tube Insertion	4 (2.2%)	9 (2.2%)
Hemodialysis	9 (5.0%)	15 (3.7%)
Enteral/Parenteral Nutrition	3 (1.7%)	2 (0.5%)
CPR	10 (5.5%)	14 (3.4%)
**Any (1+) Procedure**	37 (20.4%)	53 (13.0%)
**Decedents who are Black**	174	258
Intubation/Mechanical Ventilation	37 (21.3%)	38 (14.7%)
Tracheostomy	3 (1.7%)	4 (1.6%)
GI Tube Insertion	3 (1.7%)	11 (4.3%)
Hemodialysis	25 (14.4%)	36 (14.0%)
Enteral/Parenteral Nutrition	6 (3.5%)	4 (1.6%)
CPR	15 (8.6%)	20 (7.8%)
**Any (1+) Procedure**	62 (35.6%)	77 (29.8%)
**Decedents Ages 75+**	361	734
Intubation/Mechanical Ventilation	49 (13.6%)	80 (10.9%)
Tracheostomy	0 (0.0%)	5 (0.7%)
GI Tube Insertion	2 (0.6%)	19 (2.6%)
Hemodialysis	22 (6.1%)	38 (5.2%)
Enteral/Parenteral Nutrition	6 (1.7%)	9 (1.2%)
CPR	17 (4.7%)	29 (4.0%)
**Any (1+) Procedure**	75 (20.8%)	131 (17.9%)
**Codes Used to Identify Potentially Burdensome Care**		
Potentially Burdensome Care	CPT/HCPCS Codes	ICD-10-CM Codes
Intubation and Mechanical Ventilation	31500	0BH17EZ, 0BH18EZ, 0B717DZ, 0B718DZ, 0BH07DZ, 0BH07YZ,

© 2024 Wolff JL et al. *JAMA Internal Medicine*.

		0BH172Z, 0BH17YZ, 0BH182Z, 0BH18YZ, 0BHK7YZ, 0BHK8YZ, 0BHL7YZ, 0BHL7YZ, 0BHL8YZ, 0WHQ7YZ, 5A19, 5A1935Z, 5A1945Z, 5A1955Z, 5A12012, 5A19054
Tracheostomy	31600, 31601, 31603	0B11, 0B110F4, 0B11OZ4, 0B113F4, 0B113Z4, 0B114F4, 0B114Z4,
Gastrostomy Tube Insertion	49440, 43761	
Hemodialysis	90935, 90937	5A1D, 5A1D00Z, 5A1D60Z
Enteral or Parenteral Nutrition	43750,43246,44372,44373, 74350,43832,43830,43653, 49440,49441,49446	0DH67UZ, 0DH68UZ, 3E0G36Z
Cardiopulmonary Resuscitation	92950	5A12012, 5A19054, 5A1221Z
Potentially burdensome care codes identified by GT Wasp et al. (2020). "End-of-life quality metrics among Medicare decedents at minority-serving cancer centers: A retrospective study." Cancer Med **9**(5): 1911-1921. Subset to Maryland residents with serious illness who died 0-18 months after study entry.		

© 2024 Wolff JL et al. *JAMA Internal Medicine*.

eTable 3. Documentation of New and Updated End-of-Life Preferences by Type and Subgroup

	SHARING Choices		Control		Odds Ratios & 95% Confidence Intervals		
	Sample	Updated [1]	Sample	Updated [1]	OR (95% CI)	aOR (95% CI)[2]	aOR (95% CI)[3]
**Full Cohort: New Documentation (No Documentation in the Electronic Health Record at Baseline)**							
Advance directive (AD)	20,517	1,699 (8.3)	37,471	1,010 (2.7)	3.26 (3.01, 3.53)	3.60 (3.32, 3.90)	3.48 (3.20, 3.77)
Combined (either AD or MOLST)	18,314	2,190 (12.0)	32,321	2,130 (6.6)	1.93 (1.81, 2.05)	2.15 (2.02, 2.30)	2.03 (1.90, 2.17)
**Full Cohort: Updated Documentation (With and Without Baseline Electronic Health Record Documentation)**							
Advance directive (AD)	2,432	216 (8.9)	4,495	363 (8.1)	1.11 (0.93, 1.32)	1.10 (0.91, 1.32)	1.13 (0.94, 1.37)
Combined (either AD or MOLST)	4,635	710 (15.3)	9,645	1,060 (11.0)	1.47 (1.32, 1.62)	1.59 (1.43, 1.76)	1.59 (1.43, 1.77)
**Dementia: New Documentation (No Documentation in the Electronic Health Record at Baseline)**							
Advance directive (AD)	1,364	166 (12.2)	2,733	206 (7.5)	1.70 (1.37, 2.11)	1.80 (1.45, 2.24)	1.80 (1.44, 2.24)
Combined (either AD or MOLST)	1,087	255 (23.5)	2,084	409 (19.6)	1.26 (1.05, 1.50)	1.31 (1.09, 1.57)	1.29 (1.08, 1.55)
**Dementia: Updated Documentation (With and Without Baseline Electronic Health Record Documentation)**							
Advance directive (AD)	310	35 (11.3)	590	44 (7.5)	1.58 (0.99, 2.52)	1.26 (0.77, 2.09)	1.30 (0.79, 2.14)
Combined (either AD or MOLST)	587	145 (24.7)	1,239	197 (15.9)	1.74 (1.36, 2.21)	1.57 (1.22, 2.02)	1.59 (1.23, 2.05)
**No Dementia: New Documentation (No Documentation in the Electronic Health Record at Baseline)**							
Advance directive (AD)	19,153	1,533 (8.0)	34,738	804 (2.3)	3.67 (3.37, 4.01)	4.10 (3.75, 4.48)	3.95 (3.61, 4.32)
Combined (either AD or MOLST)	17,227	1,935 (11.2)	30,237	1,721 (5.7)	2.10 (1.96, 2.24)	2.39 (2.23, 2.56)	2.24 (2.09, 2.40)
**No Dementia: Updated Documentation (With and Without Baseline Electronic Health Record Documentation)**							
Advance directive (AD)	2,122	181 (8.5)	3,905	319 (8.2)	1.05 (0.87, 1.27)	1.07 (0.87, 1.30)	1.10 (0.89, 1.35)
Combined (either AD or MOLST)	4,048	565 (14.0)	8.406	863 (10.3)	1.42 (1.27, 1.59)	1.58 (1.40, 1.77)	1.57 (1.40, 1.77)
**Black: New Documentation (No Documentation in the Electronic Health Record at Baseline)**							
Advance directive (AD)	7,213	398 (5.5)	9,226	194 (2.1)	2.72 (2.28, 3.24)	2.88 (2.41, 3.44)	2.68 (2.23, 3.21)
Combined (either AD or MOLST)	6,583	528 (8.0)	7,995	521 (6.5)	1.25 (1.10, 1.42)	1.34 (1.18, 1.52)	1.20 (1.05, 1.37)
**Black: Updated Documentation (With and Without Baseline Electronic Health Record Documentation)**							
Advance directive (AD)	710	87 (12.3)	758	33 (4.4)	3.07 (2.03, 4.65)	2.13 (1.39, 3.26)	2.03 (1.28, 3.24)
Combined (either AD or MOLST)	1,340	183 (13.7)	1,989	153 (7.7)	1.90 (1.51, 2.38)	1.45 (1.14, 1.83)	1.39 (1.08, 1.79)
**White: New Documentation (No Documentation in the Electronic Health Record at Baseline)**							
Advance directive (AD)	11,367	1,172 (10.3)	24,024	741 (3.1)	3.61 (3.29, 3.97)	3.73 (3.39, 4.10)	3.75 (3.40, 4.13)
Combined (either AD or MOLST)	9,940	1,497 (15.1)	20,694	1,436 (6.9)	2.38 (2.20, 2.57)	2.54 (2.35, 2.75)	2.49 (2.30, 2.69)
**White: Updated Documentation (With and Without Baseline Electronic Health Record Documentation)**							
Advance directive (AD)	1,557	119 (7.6)	3,397	306 (9.0)	0.84 (0.67, 1.04)	0.90 (0.72, 1.13)	0.93 (0.74, 1.18)
Combined (either AD or MOLST)	2,984	488 (16.4)	6,727	810 (12.0)	1.43 (1.27, 1.61)	1.62 (1.43, 1.84)	1.65 (1.46, 1.88)

© 2024 Wolff JL et al. *JAMA Internal Medicine*.

**Age 75 years or older: New Documentation (No Documentation in the Electronic Health Record at Baseline)**							
Advance directive (AD)	7,953	763 (9.6)	14,660	563 (3.8)	2.66 (2.37, 2.97)	2.92 (2.60, 3.27)	2.79 (2.48, 3.13)
Combined (either AD or MOLST)	6,835	992 (14.5)	12,078	1,084 (9.0)	1.72 (1.57, 1.89)	1.90 (1.73, 2.09)	1.80 (1.63, 1.97)
**Age 75 years or older: Updated Documentation (With and Without Baseline Electronic Health Record Documentation)**							
Advance directive (AD)	1,151	97 (8.4)	2,452	202 (8.2)	1.03 (0.80, 1.32)	0.97 (0.74, 1.27)	0.99 (0.75, 1.30)
Combined (either AD or MOLST)	2,268	380 (16.8)	5,034	626 (12.4)	1.42 (1.23, 1.63)	1.51 (1.30, 1.74)	1.50 (1.30, 1.73)
**Age 74 years or younger: New Documentation (No Documentation in the Electronic Health Record at Baseline)**							
Advance directive (AD)	12,564	936 (7.5)	22,811	447 (2.0)	4.03 (3.59, 4.52)	4.43 (3.94, 4.98)	4.35 (3.86, 4.89)
Combined (either AD or MOLST)	11,479	1,198 (10.4)	20,243	1,046 (5.2)	2.14 (1.96, 2.33)	2.39 (2.19, 2.62)	2.27 (2.07, 2.48)
**Age 74 years or younger: Updated Documentation (With and Without Baseline Electronic Health Record Documentation)**							
Advance directive (AD)	1,281	119 (9.3)	2,043	161 (7.9)	1.20 (0.93, 1.53)	1.25 (0.96, 1.62)	1.32 (1.01, 1.73)
Combined (either AD or MOLST)	2,366	330 (14.0)	4,611	434 (9.4)	1.56 (1.34, 1.82)	1.71 (1.46, 2.00)	1.73 (1.47, 2.03)
**Female Sex: New Documentation (No Documentation in the Electronic Health Record at Baseline)**							
Advance directive (AD)	12,142	1,019 (8.4)	22,349	632 (2.8)	3.15 (2.84, 3.49)	3.45 (3.11, 3.82)	3.34 (3.00, 3.70)
Combined (either AD or MOLST)	10,860	1,318 (12.1)	19,239	1,317 (6.9)	1.88 (1.74, 2.04)	2.08 (1.92, 2.26)	1.97 (1.81, 2.14)
**Female Sex: Updated Documentation (With and Without Baseline Electronic Health Record Documentation)**							
Advance directive (AD)	1,433	125 (8.7)	2,708	200 (7.4)	1.20 (0.95, 1.51)	1.18 (0.92, 1.51)	1.27 (0.99, 1.63)
Combined (either AD or MOLST)	2,715	431 (15.9)	5,818	615 (10.6)	1.60 (1.40, 1.82)	1.70 (1.48, 1.95)	1.71 (1.48, 1.96)
**Male Sex: New Documentation (No Documentation in the Electronic Health Record at Baseline)**							
Advance directive (AD)	8,375	680 (8.1)	15,122	378 (2.5)	3.45 (3.03, 3.92)	3.84 (3.37, 4.38)	3.69 (3.24, 4.21)
Combined (either AD or MOLST)	7,454	872 (11.7)	13,082	813 (6.2)	2.00 (1.81, 2.21)	2.26 (2.04, 2.50)	2.12 (1.91, 2.35)
**Male Sex: Updated Documentation (With and Without Baseline Electronic Health Record Documentation)**							
Advance directive (AD)	999	91 (9.1)	1,787	163 (9.1)	1.00 (0.76, 1.31)	0.99 (0.74, 1.31)	0.97 (0.73, 1.30)
Combined (either AD or MOLST)	1,920	279 (14.5)	3,827	445 (11.6)	1.29 (1.10, 1.52)	1.43 (1.21, 1.69)	1.43 (1.21, 1.70)

1. Updated documentation of end-of-life preferences in the electronic health record by 12 months.
2. Adjusted for patient age, gender, race / ethnicity, State, and organization. Baseline documentation was added as an additional covariate in analyses of updated documentation. Race was removed as a covariate in the Black only analyses.
3. Adjusted for patient age, gender, ADI-US (2021), State, and organization. Baseline documentation was added as a covariate in analyses of updated documentation.

© 2024 Wolff JL et al. *JAMA Internal Medicine*.

eTable 4. Potentially Burdensome End-of-Life Care Among Older Decedents With Serious Illness, 6 to 18 Months After Study Entry

	SHARING Choices		Control		Unadjusted OR (95% CI)	Adjusted OR (95% CI)[1]
**Primary Analysis**	Decedent	Potentially Burdensome Care	Decedent	Potentially Burdensome Care		
Full Cohort	521	150 (28.8%)	977	204 (20.9%)	1.53 (1.20, 1.96)	1.40 (1.08, 1.81)
Dementia Status						
Dementia	181	37 (20.4%)	409	53 (13.0%)	1.73 (1.09, 2.74)	1.47 (0.89, 2.42)
No Dementia	340	113 (33.2%)	568	151 (26.6%)	1.38 (1.03, 1.84)	1.31 (0.96, 1.78)
Race and Ethnicity						
Hispanic	2	1 (50.0%)	12	1 (8.3%)	-	-
Non-Hispanic Black	174	62 (35.6%)	258	77 (29.8%)	1.30 (0.86, 1.96)	1.27 (0.81, 2.00)
Non-Hispanic White	321	82 (25.6%)	654	114 (17.4%)	1.63 (1.18, 2.24)	1.54 (1.11, 2.15)
Age						
75 years or older	361	75 (20.8%)	734	131 (17.9%)	1.21 (0.88, 1.66)	1.11 (0.80, 1.54)
74 years and younger	160	75 (46.9%)	243	73 (30.0%)	2.06 (1.36, 3.11)	2.10 (1.36, 3.24)
Sex						
Female	259	75 (29.0%)	537	102 (19.0%)	1.74 (1.23, 2.45)	1.56 (1.08, 2.25)
Male	262	75 (28.6%)	440	102 (23.2%)	1.33 (0.94, 1.88)	1.29 (0.90, 1.85)

Notes: [1]Adjusted for patient age, gender, race/ethnicity, State, and Organization (MedStar vs. JHCP). Race is not included as a covariate in the analysis of the Black subgroup.

© 2024 Wolff JL et al. *JAMA Internal Medicine*.

eFigure 1. Proportion of Older Adults Who Engaged in ACP at 19 Intervention Primary Care Practices

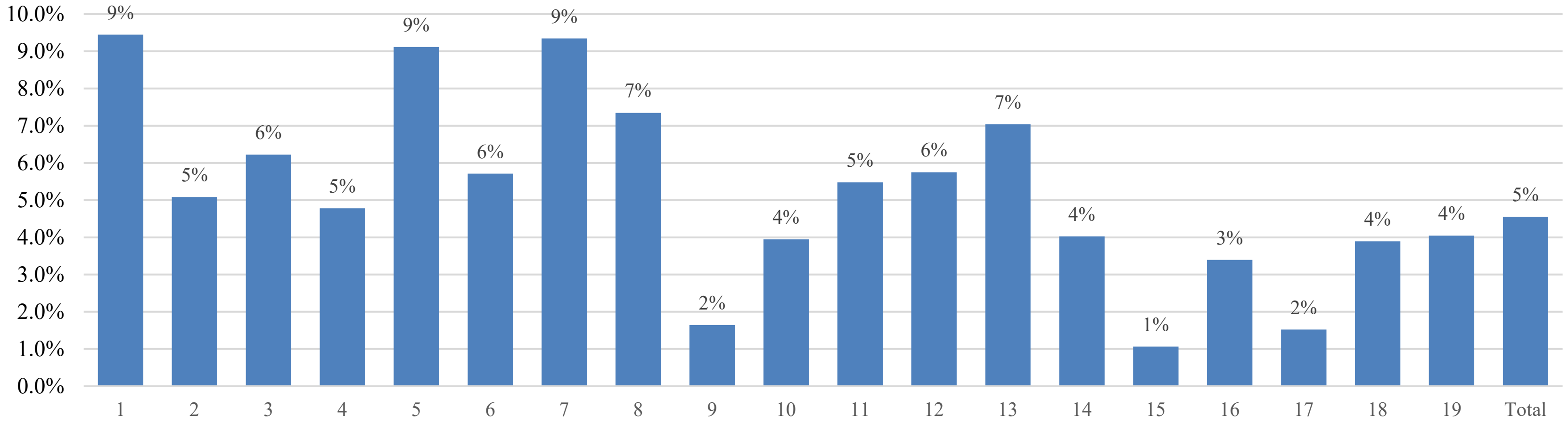

eFigure 2. New EHR-Documented End-of-Life Preferences by ACP at 19 Primary Care Practices

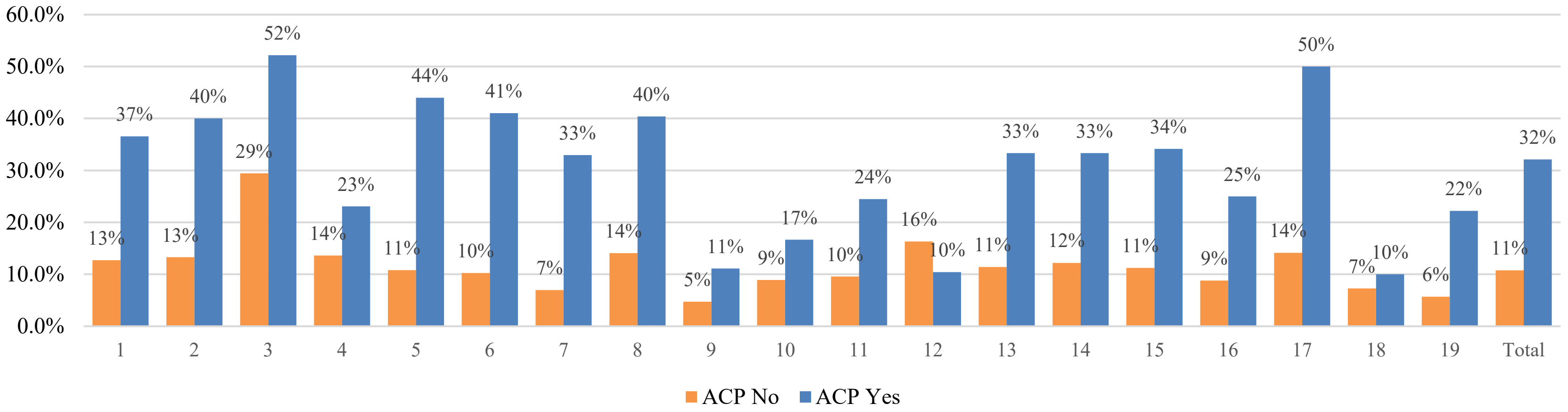

© 2024 Wolff JL et al. *JAMA Internal Medicine*.
