# Supplementary material for: Advance Care Planning, End-of-Life Preferences, and Burdensome Care: A Pragmatic Cluster Randomized Clinical Trial
Source: JAMA Intern Med. 2024 Dec 2;185(2):162–70. doi: 10.1001/jamainternmed.2024.6215 (PMC11612918; doi:10.1001/jamainternmed.2024.6215)
Supplement: Supplement 3. — Group Information. SHARING Choices Investigators [file jamainternmed-e246215-s003.pdf]

Supplemental Online Content: Nonauthor Collaborators
*First name, last name, and suffix (if applicable) are required and will appear in PubMed.

*Group Name(s): SHARING Choices Investigators							
***First Name and Middle Initial(s)**	***Last Name**	***Suffix (eg, Jr, III)**	Academic Degrees	Institution	Location (city, state/province, country)	Role or Contribution, eg, chair, principal investigator	Group (if more than 1 Group listed in the byline) and/or Subgroup (eg, Steering Committee)
Ryan	Anderson		MD	MedStar Health	Columbia, MD	Project administration	The SHARING Choices Investigators
Kimberly	Cockey			MedStar Health	Columbia, MD	Project administration	The SHARING Choices Investigators
Diane	Echavarria		MS	Johns Hopkins School of Medicine	Baltimore, MD	Project administration	The SHARING Choices Investigators
Tara	Funkhouser			Johns Hopkins Medicine	Williamsport, MD	Methodology	The SHARING Choices Investigators
Karyn Lee Carlson	Nicholson		PhD	Johns Hopkins Office of Population Health	Baltimore, MD	Project administration	The SHARING Choices Investigators
Sri	Rebala			MedStar Health	Columbia, MD	Methodology	The SHARING Choices Investigators
Talan	Zhang		MS, MPH	Johns Hopkins Bloomberg School of Public Health	Baltimore, MD	Data analyst	The SHARING Choices Investigators
