# Supplementary material for: Advance Care Planning, End-of-Life Preferences, and Burdensome Care: A Pragmatic Cluster Randomized Clinical Trial
Source: JAMA Intern Med. 2024 Dec 2;185(2):162–70. doi: 10.1001/jamainternmed.2024.6215 (PMC11612918; doi:10.1001/jamainternmed.2024.6215)
Supplement: Supplement 4. — Data Sharing Statement [file jamainternmed-e246215-s004.pdf]

Data Sharing Statement

Wolff. Advance Care Planning, End-of-Life Preferences, and Burdensome Care. *JAMA Intern Med*. Published December 02, 2024. doi:10.1001/jamainternmed.2024.6215

Data
Additional Information: ClinicalTrials.gov Identifier NCT04819191
Data available: No

Additional Information
Explanation for why data not available: The data are accessed through a data use agreement (DUA) with our two partner care delivery organizations as well as our regional health information exchange, the Maryland CRISP, which outline permitted users and storage of the dataset. The limited dataset for this research also contains sensitive patient health information which is subject to the DUAs in place at two large regional health organizations.
